# The ketone body 3-hydroxybutyrate increases cardiac output and cardiac contractility in a porcine model of cardiogenic shock: a randomized, blinded, crossover trial

**DOI:** 10.1007/s00395-025-01103-2

**Published:** 2025-04-12

**Authors:** Oskar Kjærgaard Hørsdal, Alexander Møller Larsen, Kasper Lykke Wethelund, Frederik Flyvholm Dalsgaard, Jacob Marthinsen Seefeldt, Ole Kristian Lerche Helgestad, Niels Moeslund, Jacob Eifer Møller, Hanne Berg Ravn, Roni Ranghøj Nielsen, Henrik Wiggers, Kristoffer Berg-Hansen, Nigopan Gopalasingam

**Affiliations:** 1 Department of Clinical Medicine, Aarhus University, Aarhus, Denmark; 2 Department of Cardiology, Aarhus University Hospital, Aarhus, Denmark; 3 Department of Clinical Pharmacology, Aalborg University Hospital, Aalborg, Denmark; 4 Department of Heart-, Lung-, and Vascular Surgery, Aarhus University Hospital, Aarhus, Denmark; 5 Department of Cardiology, Heart Center, Copenhagen University Hospital Rigshospitalet, Copenhagen, Denmark; 6 Department of Anesthesiology and Intensive Care, Odense University Hospital, Odense, Denmark; 7 Department of Clinical Research, University of Southern Denmark, Odense, Denmark; 8 Department of Cardiology, Gødstrup Hospital, Gødstrup, Denmark; 9 Department of Cardiology, Aarhus University Hospital, Palle Juul Jensens Boulevard 99, 8200 Aarhus N, Denmark

**Keywords:** Cardiogenic shock, 3-Hydroxy butyrate, Cardiac output, Hemodynamics, Cardiometabolic, Mitochondrial function

## Abstract

Cardiogenic shock (CS) is characterized by reduced cardiac output (CO), reduced end-organ perfusion, and high mortality. Medical therapies have failed to improve survival. The ketone body 3-hydroxybutyrate (3-OHB) enhances cardiac function in heart failure and CS. We aimed to elucidate the cardiovascular and cardiometabolic effects of 3-OHB treatment during CS. In a randomized, assessor-blinded crossover design, we studied 16 female pigs (60 kg, 5 months of age). CS was induced by left main coronary artery microsphere injections. Predefined criteria for CS were a 30% reduction in CO or mixed venous saturation (SvO_2_). Intravenous 3-OHB infusion and a matching control solution were administered for 120 min in random order. Hemodynamic measurements were obtained by pulmonary artery catheterization and a left ventricular (LV) pressure–volume catheter. Myocardial mitochondrial function was assessed using high resolution respirometry. During CS, infusion with 3-OHB increased CO by 0.9 L/min (95%CI 0.4–1.3 L/min) compared with control infusion. SvO_2_ (*P* = 0.026) and heart rate (*P* < 0.001) increased. Stroke volume (*P* = 0.6) was not altered. LV contractile function as determined by LV end-systolic elastance improved during 3-OHB infusion compared with control infusion (*P* = 0.004). Systemic and pulmonary vascular resistance decreased, and diuresis increased. LV mitochondrial function was higher after 3-OHB infusion compared with control. We conclude that 3-OHB infusion enhances cardiac function by increasing contractility and reducing vascular resistance, while also preserving myocardial mitochondrial respiratory function in a large animal model of ischemic CS. These novel findings support the therapeutic potential of exogenous ketone supplementation in CS management.

**Graphical abstract:**

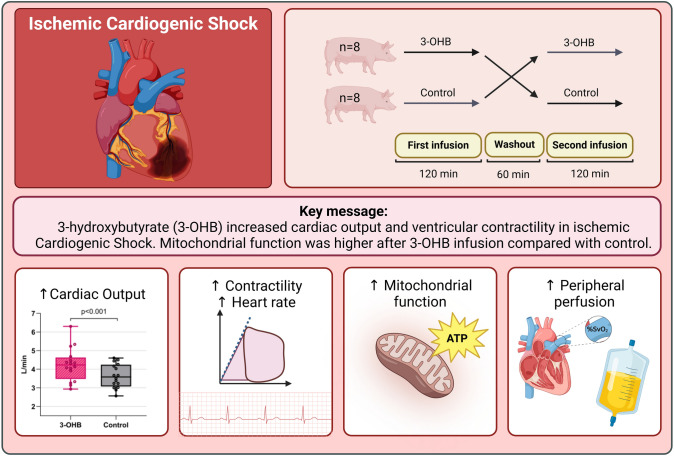

**Supplementary Information:**

The online version contains supplementary material available at 10.1007/s00395-025-01103-2.

## Introduction

Cardiogenic shock (CS) represents a multifaceted clinical syndrome frequently caused by acute myocardial infarction with an in-hospital mortality of 30–50% [65]. Key pathophysiological features in CS encompass depression of myocardial contractility leading to impaired cardiac output (CO), compromised end-organ perfusion, and cardiovascular collapse. Present strategies focus on improving the compromised hemodynamic status and often involve inotropes, vasopressors, and mechanical circulatory support [37]. Yet, no medical treatment has improved the survival rate of patients with CS [63, 65], stressing the need for novel treatment options.

Modulation of circulating levels of ketone bodies has been suggested as a promising treatment option in critical care settings. Elevating circulating levels of ketones have improved cardiac function in patients with CS and decreases cerebral injury biomarkers in a porcine cardiac arrest model [2, 5]. Given the innate cardiac ability to efficiently utilize ketone bodies, increased circulating ketone body levels could confer positive effects on myocellular metabolism [40, 60]. Infusion with the ketone body 3-hydroxybutyrate (3-OHB) increases CO in patients with heart failure without compromising cardiac energy efficiency [44]. 3-OHB provides an efficient and alternative energy substrate for myocardial metabolism, and elevating 3-OHB holds promise as a treatment during cardiometabolic stress [35, 43]. In healthy rats, 3-OHB has been demonstrated to enhance cardiac contractility and induce vasorelaxation ex vivo [25]. In the setting of CS, where myocardial energy demand exceeds supply, treatment with 3-OHB may hold potential as a critical energy source to support myocardial energy production.

Still, the mechanistic hemodynamic insights in vivo within the context of ischemic CS remain unexplored. In this study, we aimed to investigate the direct impact of 3-OHB on hemodynamic and cardiometabolic function in a controlled large-animal model of ischemic CS [24, 38, 57], offering novel insights beyond previous trials. By utilizing state-of-the-art pressure–volume loop (PV) analysis and high-resolution myocardial respirometry, we aimed to comprehensively characterize the cardiac response of ketone treatment in CS. We hypothesized that treatment with 3-OHB increases CO and perfusion during CS through increased contractility and vasorelaxation while enhancing mitochondrial respiratory function.

## Methods

### Experimental protocol

The pigs were initially catheterized following anesthesia and intubation procedures. Baseline measurements were performed followed by slow injections of polyvinyl alcohol microspheres (Contour™, Boston Scientific, USA) in the left main coronary artery to promote LV dysfunction through irreversible obstruction of microcirculation[28–30, 38]. Hemodynamic parameters were allowed to stabilize for 3 min following each injection before additional injection of microspheres. CS was defined as a 30% reduction in CO or mixed venous saturation (SvO_2_) compared with baseline. When CS criteria were met, a 1-h no-touch period was introduced prior to study interventions was initiated to allow the pig to demonstrate hemodynamic stability without rapid spontaneous improvement or deterioration. This approach was based on unpublished data from our laboratory, demonstrating that a 1-h no-touch period ensured hemodynamic stability in the animals (Table 1, Supplemental Table 1).

**Table 1:** Hemodynamic characteristics in healthy state and in shock

*N* = *16*	Baseline	Shock	*P *value
Hemodynamic parameters			
CO, L/min	4.5 ± 0.9	3.1 ± 0.8	** < 0.001**
SvO_2_, %	55.0 ± 8.1	37.1 ± 6.5	** < 0.001**
MAP, mmHg	84 ± 15	68 ± 10	** < 0.001**
CPO, W	0.85 ± 0.26	0.47 ± 0.18	** < 0.001**
PaPi	3.3 (2.3; 4.3)	2.3 (1.5; 3.3)	0.421
SV, mL	76 ± 15	48 ± 22	** < 0.001**
HR, bpm	63 (53; 68)	68 (56; 74)	0.051
mPAP, mmHg	19 ± 5	22 ± 5	0.267
RAP, mmHg	6 ± 3	7 ± 3	0.430
PAWP, mmHg	8 ± 3	14 ± 4	**0.004**
SVR, dyn*cm^5^	1413 ± 291	1695 ± 495	0.075
PVR, dyn*cm^5^	204 ± 101	210 ± 495	0.121
P(v-a)CO_2_, kPa	1.39 ± 0.32	1.66 ± 0.36	0.182
Pressure–volume parameters			
LVESV, mL	109 ± 32	115 ± 33	0.559
LVESP, mmHg	99 ± 14	84 ± 16	**0.001**
LVEDV, mL	190 ± 39	172 ± 42	0.249
LVEDP, mmHg	15 ± 6	20 ± 7	**0.008**
LVEF, %	50 (37; 64)	36 (33; 43)	**0.034**
Ees, mmHg/mL	1.45 ± 0.56	1.11 ± 0.31	**0.043**
Ea, mmHg/mL	1.66 ± 0.91	1.92 ± 0.93	0.135
Ea/Ees ratio	1.42 ± 1.00	1.89 ± 0.68	0.534
Stroke work, mmHg*mL	6,599 ± 3,073	3,678 ± 2,094	** < 0.001**
Potential energy, mmHg*mL	3,806 ± 1,501	3,441 ± 1,398	0.304
Pressure–Volume Area, mmHg*mL	10,351 ± 3,187	7,153 ± 3,074	** < 0.001**
Cardiac mechanical efficiency, %	60 (50; 70)	44 (42; 55)	**0.007**

Hemodynamic parameters and pressure–volume parameters at baseline and at shock (following one-hour no touch period after reaching prespecified cardiogenic shock criteria) where the first intervention was initiated. Data are expressed as mean ± SD or median (interquartile range). *P*-value indicates paired T-test for normally distributed data or Wilcoxon signed rank test for non-normally distributed data comparing data from healthy state with shock state. **Bold** values indicate *P* < 0.05

CO = cardiac output, CPO = cardiac power output, PaPi = pulmonary artery pulsatile index, HR = heart rate, SV = stroke volume, SvO_2_ = mixed venous saturation, P(v-a)CO_2_ = veno-arterial carbon dioxide difference, MAP = mean arterial pressure, mPAP = mean pulmonary artery pressure, RAP = right atrium pressure, PAWP = pulmonary artery wedge pressure, SVR = systemic vascular resistance, PVR = pulmonary vascular resistance, Ea = arterial elastance, Ees = end-systolic elastance (the slope of the end-systolic pressure–volume relationship), LVESV = end-systolic volume, LVEDV = end-diastolic volume, LVEF = left ventricular ejection fraction. Cardiac mechanical efficiency is calculated as the ratio between stroke work and pressure–volume area

**Fig. 1:**
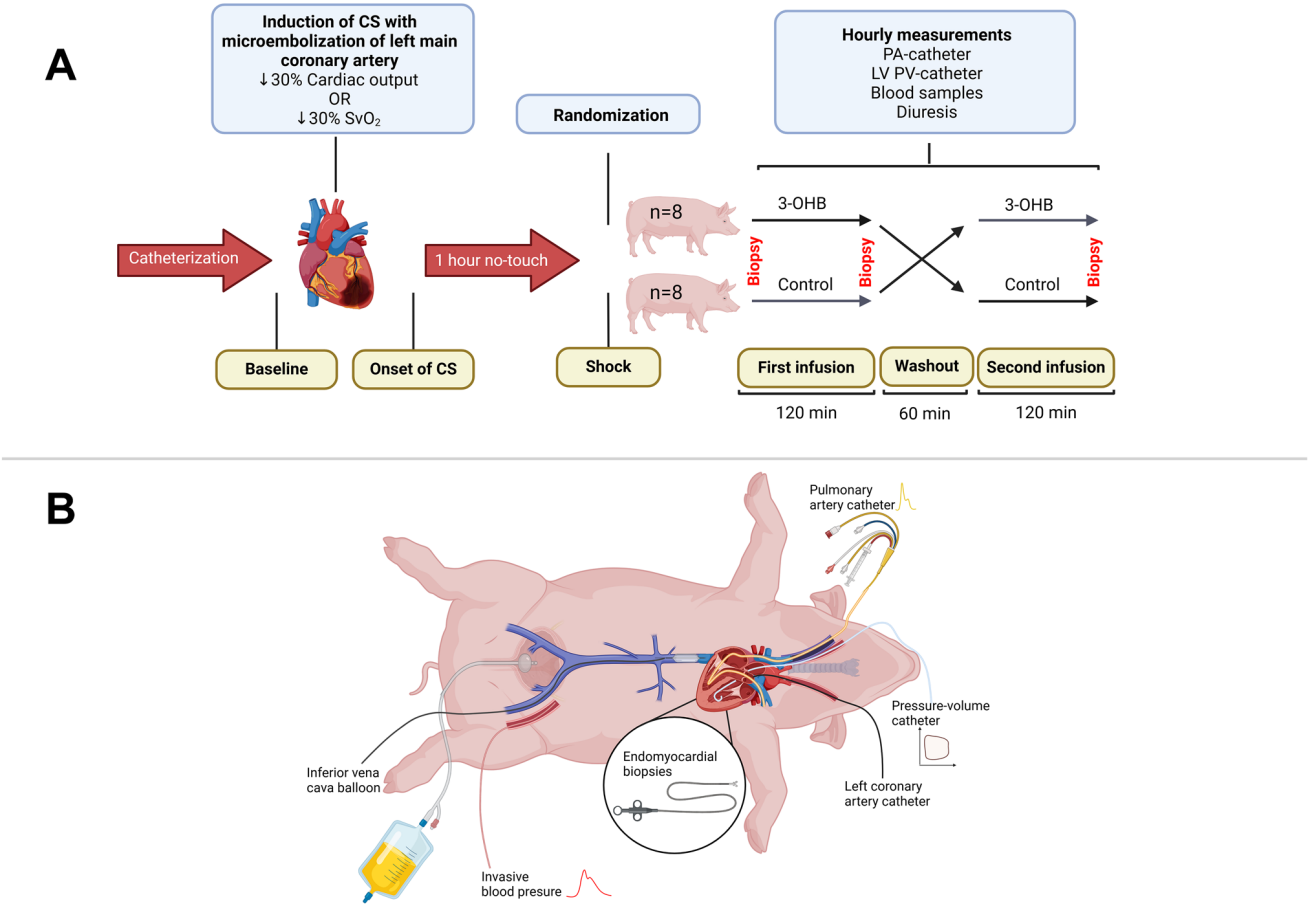
Study Design and Instrumentation. **A** Upon catheterization, cardiogenic shock (CS) was induced using repeated injections of microspheres into the left main coronary artery. CS was considered present with 30% reduction in mixed venous saturation (SvO_2_) or cardiac output measured with bolus thermodilution. The pigs then underwent a one-hour no-touch period to enable hemodynamic stabilization before study intervention. All pigs (*N* = 16) were randomized to receive a two-hour infusion of 3-OHB and control in random order, separated by a one-hour washout period during which all pigs received isotonic saline. Hourly hemodynamic measurements were performed during the study period. **B** A pulmonary artery (PA) catheter was placed through the jugular vein for hemodynamic assessment. A pressure–volume (PV) admittance catheter was in the left ventricle (LV) through the right carotid artery. A coronary 3.5 L guide catheter was inserted through the left carotid artery into the left main coronary artery. The same vascular access was used for LV endocardial biopsies. A balloon catheter was inserted through a femoral vein. The balloon was placed on diaphragm level. Invasive blood pressure was measured continuously using a fluid filled catheter in the femoral artery

In a randomized, assessor-blinded crossover design (Fig. 1A), sixteen pigs were randomized into two groups (*n* = 8 receiving 3-OHB before crossover to control, and *n* = 8 receiving control before crossover to 3-OHB) using computer-generated randomization. The pigs received 120 min intravenous infusion with 2.9 ml/kg/h 3-OHB (75 g/L, racemic mixture) (Sodium 3-hydroxybutyrate, Gold Biotechnology Inc., St. Louis, MO, USA) followed by 2.9 ml/kg/h matched control (equimolar NaCl solution) or vice versa, separated by a 1-h washout period. Our previous studies have demonstrated that this dosage of 3-OHB raises plasma levels to a target range, producing a measurable cardiovascular effect [20]. Also, this design ensures a stable cardiac dysfunction without active treatment (Supplemental Fig. 1). Pigs that died before randomization were replaced.

The primary endpoint was CO through 120 min of 3-OHB infusion compared with 120 min of control infusion. All secondary endpoints were compared similarly.

### Animals

In this prospective experimental study, 60-kg Danish Landrace pigs with an age of 5 months were included following the requisite authorization from the Danish national Animal Experiment Inspectorate (*Hemodynamic Optimization with Carbonates,* Permit no: 2023-15-0201–01466*.* Issue date: 19/06–2023). The treatment and ethical oversight of the animals strictly adhered to established animal welfare protocols and regulatory standards stipulated by both Danish and European legislation. The methodologies and animal handling conformed to the EU Directive 2010/63/EU pertaining to animal experimentation. Upon the end of the study, all animals were euthanized with a lethal intravenous dose of ≥ 0.25 mg/kg pentobarbital (*Euthanimal, Scanvet, Denmark*)*.* The study followed the ARRIVE 2.0 guidelines.

### Animal preparation

The study applied a procedure to minimize stress and increase refinement in the care of the pigs. Initially, pigs received sedation on the farm via intramuscular injection of a commonly used anesthetic mix (*Zoletil 50 Vet, Virbac, Denmark*) prior to transportation to the laboratory facility. The pigs were intubated and immediately placed under invasive positive-pressure ventilation. Anesthesia maintenance entailed a continuous infusion of propofol (3.5 mg/kg/h) and fentanyl (15 µg/kg/h). Ventilation parameters included a tidal volume of 8 ml/kg with respiratory rate adjustments to maintain end-tidal CO_2_ between 4.5 and 5.5 kPa. PEEP was set to 5.0 cmH_2_O. Prior to baseline measurements, fractional inspired oxygen was adjusted to the lowest level to maintain PaO_2_ within 11–13 kPa. Heart rate (HR) was continuously monitored. During the first hour of instrumentation, the pigs received a continuous infusion of 10 mL/kg/h isotonic saline (1 L) to maintain euvolemia. Afterwards, all pigs received a continuous infusion of 2.9 mL/kg/h isotonic saline until the start of the study period. Invasive catheterizations were performed as shown in Fig. 1B. If the pigs developed arrhythmias during instrumentation, synchronized cardioversion was applied accordingly.

### Pulmonary artery catheterization

A pulmonary artery (PA) catheter (Swan Ganz, Edwards Lifesciences, Irvine, CA, USA) was placed through the internal jugular vein under pressure guidance (Fig. 1B). Correct placement was confirmed by fluoroscopy. CO was measured with the bolus thermodilution method, using a Vigilance box (Edwards Vigilance VGS, Edwards Lifesciences, Irvine, CA, USA*)* and averaged over three consecutive measurements with less than 10% variation. Right atrial pressure (RAP), mean PA pressure (mPAP), and PA wedge pressure (PAWP) were measured hourly. Stroke volume (SV = CO/HR), systemic (SVR = 80 × (MAP-RAP)/CO) and pulmonary vascular resistance (PVR = 80 × (mPAP-PAWP)/CO), cardiac power output (CPO = (mean arterial blood pressure × CO)/451) [14], and PA pulsatility index (PaPi = [PA pulse pressure]/RAP) [33] were calculated. SvO_2_ was measured in a mixed venous blood gas from the distal port of the PA catheter using a blood gas analyzer (ABL90 Flex, Radiometer, Denmark*)*. Mean arterial blood pressure (MAP) was assessed using an intravascular fluid-filled pressure catheter in the femoral artery. HR was monitored using a three-lead continuous electrocardiogram.

### Left ventricular pressure–volume catheter

A PV admittance catheter (Transsonic, Ithaka, NY, USA*)* was inserted into the LV through the carotid artery using fluoroscopy. The catheter was fixed and left untouched for the whole study period. PV recordings were initially pressure calibrated and subsequently volume calibrated by utilizing SV as calculated from the PA catheter. All data were obtained during apnea. PV measurements conducted hourly in LabChart 8 Pro (AD Instruments, Sydney, Australia) for blinded offline analysis. A transfemoral occluding balloon *(*Edwards Transfemoral Balloon Catheter, Edwards Lifesciences, Irvine, CA, USA) was placed in the inferior vena cava at the diaphragm level. At baseline, a balloon occlusion of the inferior vena cava was performed to acquire the theoretical ventricular volume when no pressure is generated (*V*_0_) [49, 55]. Arterial elastance (Ea) was estimated as the slope of the line intersecting at LV end-diastolic volume (LVEDV) and LV end-systolic pressure (LVESP). Ventriculo-arterial coupling (VA-coupling) was calculated as Ea/Ees. Preload was assessed using LVEDV and end-diastolic pressure (LVEDP). Additional hemodynamic parameters were assessed, including LV end-systolic volume (LVESV), LV ejection fraction (LVEF), and LV diastolic time constant (tau). End-systolic elastance was estimated as (Ees = LVESP/[LVESV-*V*_0_])) and defined as the slope of the end-systolic PV relationship (ESPVR).

LV mechano-energetic parameters were calculated. Stroke work (SW), which is the area inside the PV-loop and represents the ventricular energy delivered to the arterial system for maintaining forward blood flow, was calculated by the Labchart 8 Pro software upon blinded manual approvement of PV loops. Potential energy (PE), which is the area of the pressure–volume diagram bounded by the ESPVR, the end-diastolic PV relationship and end-systolic portion of the PV loops, was estimated as LVESP × (LVESV-V0)/2 [18]. PE represents the ventricular energy that is dissipated as heat during isovolumetric relaxation. Hence, the total mechanical energy during one cardiac cycle is the sum of both SW and PE, resulting in the pressure–volume area (PVA). LV cardiac mechanical efficiency (SW/PVA), which represents the ratio of generated work translated into pumping blood. Cardiac work (CW = PVA × heart rate) were calculated.

### Left main coronary artery catheterization

The left main coronary artery was catheterized under fluoroscopy guidance with a JL 3.5 catheter (Launcher, Medtronic Inc., Minneapolis, MN, USA) through the left carotid artery (Supplemental Video 1). The left anterior descending artery and the left circumflex artery were identified during contrast injection. The catheter was then fixated and used to inject microemboli into the left coronary main artery. The catheter was removed when CS criteria were met.

### Endomyocardial mitochondrial respirometry

A flexible biopsy forceps (Jawz™, Argon Medical Devices, USA) was advanced through the left carotid artery to the LV under fluoroscopic guidance. Endomyocardial biopsies were taken from the interventricular septum. Mitochondrial respiratory capacity was measured (Oxygraph 2 K, Oroboros Instruments, Innsbruck, Austria) as previously described [53]. The following substrate protocol was used to evaluate mitochondrial respiration: Glutamate (10 mmol/L) and Malate (2 mmol/L). Subsequent addition of ADP (5 mmol/L) allows complex I mediated respiration with electron flow through the mitochondrial ATPase. Succinate (10 mmol/L) was added to stimulate maximal respiration with electron flow through complex I + II (OXPHOS). Oligomycin (complex V inhibitor) (2 μg/mL) was added to evaluate state 4o leak respiration. Final addition of rotenone (complex I inhibitor) (0.5 μmol/L) and antimycin A (complex III inhibitor) (2.5 mmol/l) allows measurement of residual oxygen consumption. Our aim was to assess the intrinsic oxidative phosphorylation capacity of the mitochondria. By bypassing upstream metabolic steps by using this standard protocol [11, 32] for analysis of permeabilized fibers and focusing on complexes I and II, the maximum capacity of the electron transport system rather than the immediate effect of specific in vivo substrates is quantified. This approach ensures that any observed mitochondrial effects reflect mitochondrial damage or preservation induced by prior in vivo intervention exposure rather than acute substrate-driven alterations in respiration. To avoid any O_2_ limitations to respiration the chambers were hyperoxygenated and all measurements were carried out in duplicate. For a detailed protocol, please refer to Supplemental Material S1.

Endomyocardial biopsies were obtained at three timepoints during CS: (1) Shock state, (2) At the end of the first infusion period, and (3) At the end of the second infusion period.

### Biochemistry and fluid balance

Arterial and mixed venous blood samples from the femoral and pulmonary arteries, respectively, were obtained simultaneously at baseline and every hour during the entire study period (Fig. 1A). Arterial plasma levels of high-sensitivity troponin I (hs-TnI) were analyzed in batch using a sandwich immunometric high-sensitivity method (Atellica IM, Siemens Healthineers, Germany). Lactate, glucose, electrolytes, and acid–base parameters (pH, PaCO_2_, HCO_3_^−^) were analyzed immediately after sampling with the blood gas analyzer. The veno-arterial CO_2_ tension difference (P(v-a)CO_2_) was calculated as a measure of peripheral tissue perfusion [26, 36].

### Statistical methods

The standard deviation of CO (primary endpoint), measured using thermodilution in untreated pigs with CS in this experimental model, is 0.4 L/min (unpublished data from our research facility). By enrolling 16 pigs (8 per group), an effect size of 0.6 L/min would be detected with a power of 80% and a two-sided significance level of 5%. Data were analyzed for normal distribution with qq plots and histograms. Normally and non-normally distributed variables are presented as mean ± standard deviation (SD) and median with interquartile range (IQR), respectively. Continuous data were analyzed using a linear mixed effects model to compare the effect of 3-OHB with the control. Residuals were tested for normality and parameters were log-transformed accordingly. Treatment, time, treatment-by-time interaction, period, and treatment sequence were defined as fixed effects, whereas animals were selected as random effects to account for interindividual variability and allow each animal to serve as its own control. The mean treatment effects of 3-OHB infusion versus the control is presented with 95% confidence intervals (CI). Statistical significance was set at a two-tailed *P* value < 0.05. Statistical analyses were conducted in the *R* software (Version 4.2.1, Rstudio, PBC) and graphics were constructed using Prism (Version 8.4.2, GraphPad, San Diego, CA, USA) or the *R* software*.*

## Results

### Induction of cardiogenic shock

A total of 16 animals with CS were included (Table 1, Supplemental Table 1). One pig, receiving control as the first infusion, died after 120 min of control infusion from incessant malignant ventricular arrhythmias. No pigs receiving 3-OHB as infusion experienced ventricular arrhythmias. One pig died before randomization during the 1-h no-touch period. Both pigs were replaced 1:1 according to the predefined study plan. After induction of CS, at the end of the 1-h no-touch period, CO was reduced from 4.5 ± 0.9 L/min in healthy state to 3.1 ± 0.8 L/min (31% reduction, *P* < 0.001) and SvO_2_ decreased from 55 ± 8% to 37 ± 7% (31% reduction, *P* < 0.001; Table 1). The immediate hemodynamic response to CS was consistent with the one-hour measurements, as detailed in Supplemental Table 1. Arterial lactate levels increased from 1.2 ± 0.5 to 1.9 ± 0.8 mmol/L (*P* = 0.011). CPO and MAP were decreased in CS while LV filling pressures (LVEDP and PAWP) were increased, accompanied by decreased SV. CS increased Ea while Ees decreased resulting in lowered mechano-energetic function. This was evidenced by lower SW and subsequent decreased cardiac mechanical efficiency and Ea/Ees ratio.

### Hemodynamic effects of 3-OHB infusion

**Fig. 2:**
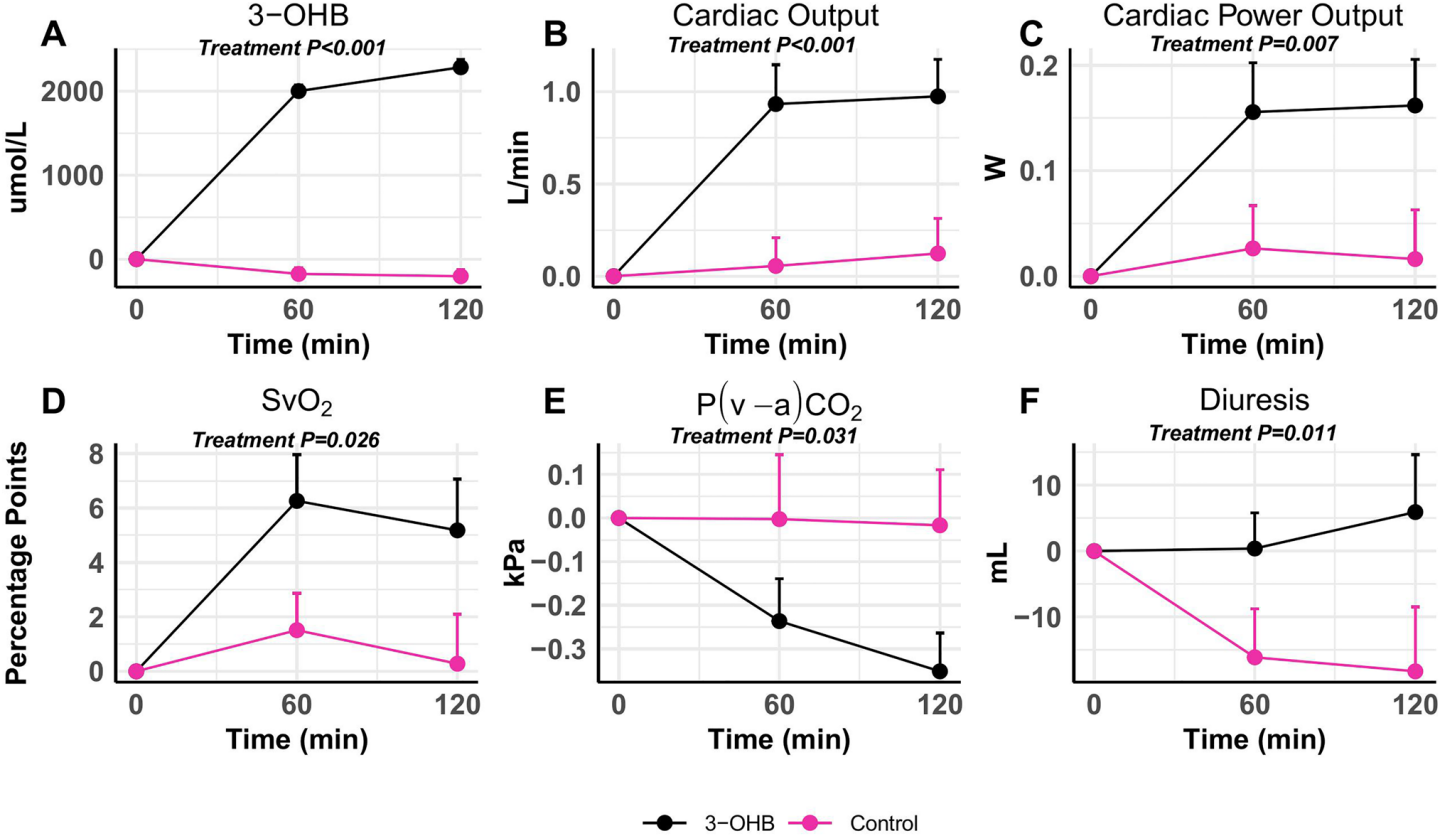
Changes in cardiac output and indices of perfusion during 3-OHB and control infusion. Temporal changes in arterial 3-hydroxybutyrate (3-OHB) concentration, cardiac output, cardiac power output, mixed venous saturation (SvO_2_), venoarterial CO_2_-difference (P(v-a)CO_2_, and diuresis. 16 animals were studied. Data are shown as mean + SEM change from start of infusion. *P*-values are derived from the linear mixed model analysis

**Fig. 3:**
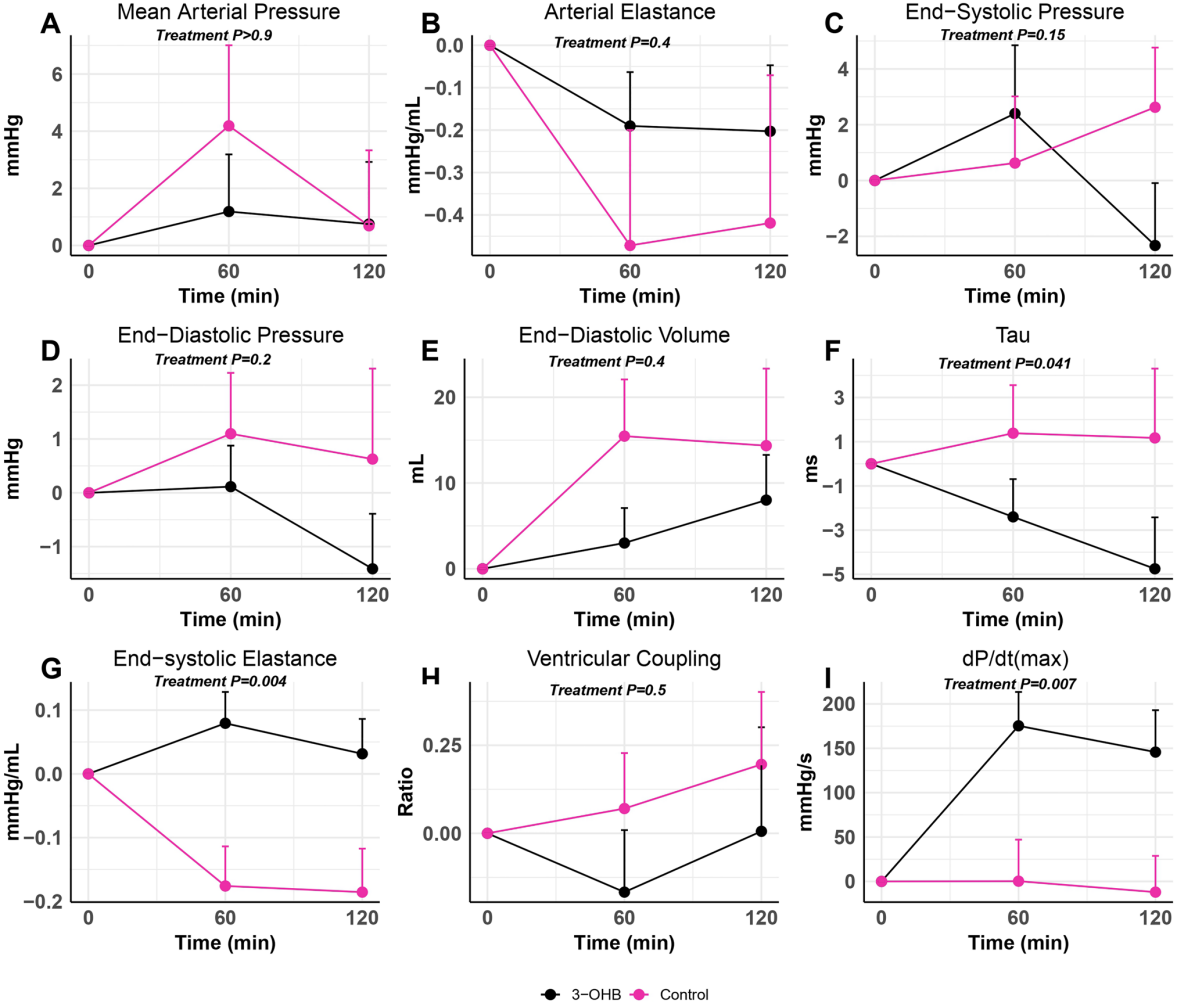
Changes in additional hemodynamic parameters during 3-OHB and control infusion Changes in afterload (**A**–**C**): mean arterial blood pressure, arterial elastance and end-systolic pressure. Changes in preload and filling parameters (**D**–**F**): end-diastolic pressure, end-diastolic volume and pulmonary artery wedge pressure. Changes in contractility (**G**–**I**): end-systolic elastance, ventricular coupling and d*P*/d*t*(max). 16 animals were studied

Infusion with 3-OHB raised circulating levels of 3-OHB by 2.5 mmol/L (95% CI 2.3 to 2.7 µmol/L, *P* < 0.001; Table 2 and Fig. 2). In parallel, CO increased by 0.9 L/min (95% CI 0.4 to 1.3 L/min, *P* < 0.001) during 3-OHB infusion compared with the control (Table 3, Figs. 2, 3. and 4, Supplemental Fig. 2). The response did not differ between treatment sequence allocation (*P* = 0.994; Supplemental Fig. 3). No interaction of treatment sequence for CO was observed (*P* = 0.8). CPO and SvO_2_ improved while P(v-a)CO_2_ decreased during 3-OHB infusion compared with control infusion. Infusion with 3-OHB increased HR by 21 bpm (95% CI 11 to 32 bpm, *P* < 0.001) compared with control, while SV, MAP, and mPAP did not differ significantly. Thus, SVR and PVR were decreased. PaPi remained unchanged during both infusions. Diuresis increased by 23 mL/h (95% CI 3 to 63 mL/h, *P* = 0.011) during 3-OHB infusion compared with control infusion (Table 3, Fig. 2). 3-OHB infusion caused no ventricular arrhythmias. Intermittent supraventricular tachycardias with no apparent hemodynamic significance were developed in four animals during 3-OHB infusion and in two animals during control infusion.

**Table 2:** Changes in biomarkers during shock: effects of infusion of 3-OHB and control infusion

	Control		3-OHB		Linear mixed model		
	Start of treatment (*n* = 16)	After 120 min	Start of treatment (*n* = 16)	After 120 min	Pairwise comparison (95% CI)	*P* value	*P*-value for interaction of treatment sequence
3-OHB, mmol/L	0.0 ± 0.0	0.1 ± 0.0	0.0 ± 0.0	2.3 ± 0.4	2.5 (2.3 to 2.7)	** < 0.001**	0.083
Lactate, mmol/L	1.7 (1.2; 2.5)	1.1 (0.9; 1.5)	1.2 (0.9; 1.8)	1.5 (1.3; 2.2)	0.4 (0.2 to 0.8)	** < 0.001**	**0.010**
Free Fatty Acids, mmol/L	0.35 (0.26; 0.41)	0.32 (0.26; 0.37)	0.29 (0.25; 0.34)	0.14 (0.10; 0.23)	− 0.03 (− 0.05 to − 0.01)	**0.034**	0.2
Glucose, mmol/L	6.4 ± 1.9	6.5 ± 1.0	6.8 ± 1.2	6.2 ± 0.7	− 0.6 (− 1.4 to 0.2)	0.12	> 0.9
pH	7.48 ± 0.06	7.50 ± 0.05	7.45 ± 0.03	7.50 ± 0.04	0.06 (0.04 to 0.07)	** < 0.001**	0.2
Bicarbonate, mmol/L	30.7 ± 3.6	31.0 ± 3.3	27.8 ± 1.8	34.0 ± 2.1	5.4 (4.7 to 6.0)	** < 0.001**	**0.026**
Potassium, mmol/L	3.8 ± 0.4	4.1 ± 0.3	4.1 ± 0.3	3.8 ± 0.3	− 0.6 (− 0.7 to − 0.5)	** < 0.001**	0.3
Sodium, mmol/L	140 ± 2	145 ± 2.7	140.0 ± 2.0	144.0 ± 1.9	− 0.4 (− 1.0 to 0.3)	0.3	> 0.9
Hemoglobin, mmol/L	6.2 ± 0.7	5.9 ± 0.64	6.0 ± 0.7	6.1 ± 0.67	0.25 (0.06 to 0.44)	**0.012**	0.5
Hematocrit, %	31 ± 4	29 ± 3.2	30 ± 3	30 ± 3.3	1.2 (0.26 to 2.1)	**0.014**	0.5
Troponin I, ng/L	26,550 (1,500; 92,040)	107,670 (13,665; 216,315)	12,390 (3,045; 31,965)	54,810 (31,875; 138,690)	21,777 (− 4,636 to 246,158)	0.6	**0.04**

Biomarkers after 120 min of each infusion. Data are expressed as mean ± SD or median (interquartile range). Changes in biomarkers during 3-hydroxybutyrate (3-OHB) compared with control through each 120-min treatment period were assessed using a repeated measurements linear mixed model. **Bold** values indicate *P* < 0.05

**Table 3:** Hemodynamic parameters during shock: effects of 3-OHB and control infusion

	Control		3-OHB		Linear mixed model		
	Start of treatment (*n* = 16)	After 120 min	Start of treatment (*n* = 16)	After 120 min	Pairwise comparison (95% CI)	*P* value	*P* value for interaction of treatment sequence
Hemodynamic parameters							
CO, L/min	3.5 ± 0.9	3.6 ± 0.7	3.3 ± 0.8	4.2 ± 0.9	0.9 (0.4 to 1.3)	** < 0.001**	0.8
SvO_2_ (%)	38.4 ± 7.2	38.0 ± 6.1	38.0 ± 6.0	42.0 ± 8.5	4.7 (0.7 to 8.8)	**0.026**	0.3
MAP (mmHg)	68 ± 12	68 ± 9	67 ± 8	68 ± 12	0 (− 6 to 6)	> 0.9	0.2
CPO (W)	0.53 ± 0.19	0.54 ± 0.11	0.49 ± 0.14	0.65 ± 0.22	0.14 (0.04 to 0.25)	**0.007**	0.4
PaPi (W)	2.7 (1.8; 3.6)	3.1 (2.1; 4.3)	2,7 (1.7; 4.4)	3.3 (2.1; 3.7)	0.2 (− 0.4 to 1.6)	0.6	0.5
SV (mL)	45 ± 22	42 ± 14	47 ± 18	46 ± 17	2 (− 5 to 9)	0.6	**0.033**
HR (bpm)	89 ± 38	94 ± 32	74 ± 18	100 ± 30	21 (11 to 32)	** < 0.001**	**0.013**
mPAP (mmHg)	22 ± 6	24 ± 6	23 ± 4	22 ± 5	− 2 (− 5 to 0)	0.080	0.4
RAP (mmHg)	6 ± 3	6 ± 3	6 ± 3	6 ± 3	0 (− 1 to 1)	0.6	0.5
PAWP (mmHg)	14 ± 5	14 ± 5	14 ± 4	13 ± 5	− 1 (− 3 to 1)	0.3	0.4
SVR (dyn/s/cm^5^)	1,495 ± 528	1,441 ± 415	1,577 ± 447	1,208 ± 290	− 331 (− 508 to − 153)	** < 0.001**	0.5
PVR (dyn/s/cm^5^)	210 ± 114	243 ± 130	224 ± 92	175 ± 93	− 72 (− 142 to − 2)	**0.047**	> 0.9
P(v-a)CO_2_, kPa	1.61 ± 0.37	1.60 ± 0.32	1.68 ± 0.27	1.40 ± 0.27	− 0.30 (− 0.57 to − 0.03)	**0.031**	0.2
Pressure–volume parameters							
LVESV, mL	117 ± 37	129 ± 34	123 ± 30	129 ± 38	− 10 (− 21 to 1)	0.071	0.4
LVESP, mmHg	83 ± 16	85 ± 15	82 ± 12	80 ± 17	− 5 (− 10 to 2)	0.15	0.2
LVEDV, mL	173 ± 48	187 ± 45	177 ± 40	186 ± 51	− 7 (− 23 to 10)	0.4	0.2
LVEDP, mmHg	20 ± 9	21 ± 7	21 ± 7	20 ± 8	− 2 (− 5 to 1)	0.2	0.8
LVEF, %	40 ± 12	37 ± 10	40 ± 18	44 ± 19	6 (1 to 11)	**0.020**	0.5
dP/dt(max), mmHg/s	1,228 ± 277	1,213 ± 232	1,206 ± 306	1,362 ± 361	167 (49 to 284)	**0.007**	0.6
Ees, mmHg/mL	1.12 ± 0.35	0.93 ± 0.21	1.01 ± 0.29	1.04 ± 0.27	0.22 (0.08 to 0.37)	**0.004**	0.2
Ea, mmHg/mL	2.5 ± 1.7	2.0 ± 1.1	2.0 ± 1.1	1.8 ± 1.1	0.2 (− 0.3 to 0.8)	0.4	0.3
Ea/Ees ratio	1.8 ± 1.0	2.0 ± 1.2	2.0 ± 0.7	2.0 ± 1.5	− 0.2 (− 0.7 to 0.3)	0.5	0.7
Cardiac mechanical efficiency, %	49 ± 13	46 ± 12	47 ± 9	50 ± 13	6 (1 to 11)	**0.014**	0.6
Stroke work, mmHg*mL	3,737 ± 2,201	3,847 ± 2,211	3,627 ± 1,727	4,266 ± 2,360	570 (− 201 to 1,342)	0.2	0.14
Pressure-Volume Area, mmHg*mL	7249 (5076; 9681)	7,666 (5,542; 8,906)	7583 (5359; 8139)	8,222 (6,071; 8,654)	− 511 (− 1,037 to 859)	0.4	0.098
CW, mmHg*L	566 ± 255	719 ± 387	507 ± 194	775 ± 359	88 (− 69 to 246)	0.3	> 0.9
Tau, ms	50 ± 11	52 ± 8	54 ± 11	48 ± 8	− 6 (− 12 to 0)	**0**.**041**	0.8
Diuresis							
Diuresis, mL/h	47 (36; 63)	30 (23; 37)	34 (26;45)	42 (36; 50)	23 (3 to 63)	**0.011**	0.9

Hemodynamic parameters after 120 min of each infusion. Data are expressed as mean ± SD or median (interquartile range). Hemodynamic changes during 3-hydroxybutyrate (3-OHB) infusion compared with control through each 120-min treatment period were assessed using a repeated measurements linear mixed model. **Bold** values indicate *P* < 0.05

Abbreviations as in Table 1

**Fig. 4:**
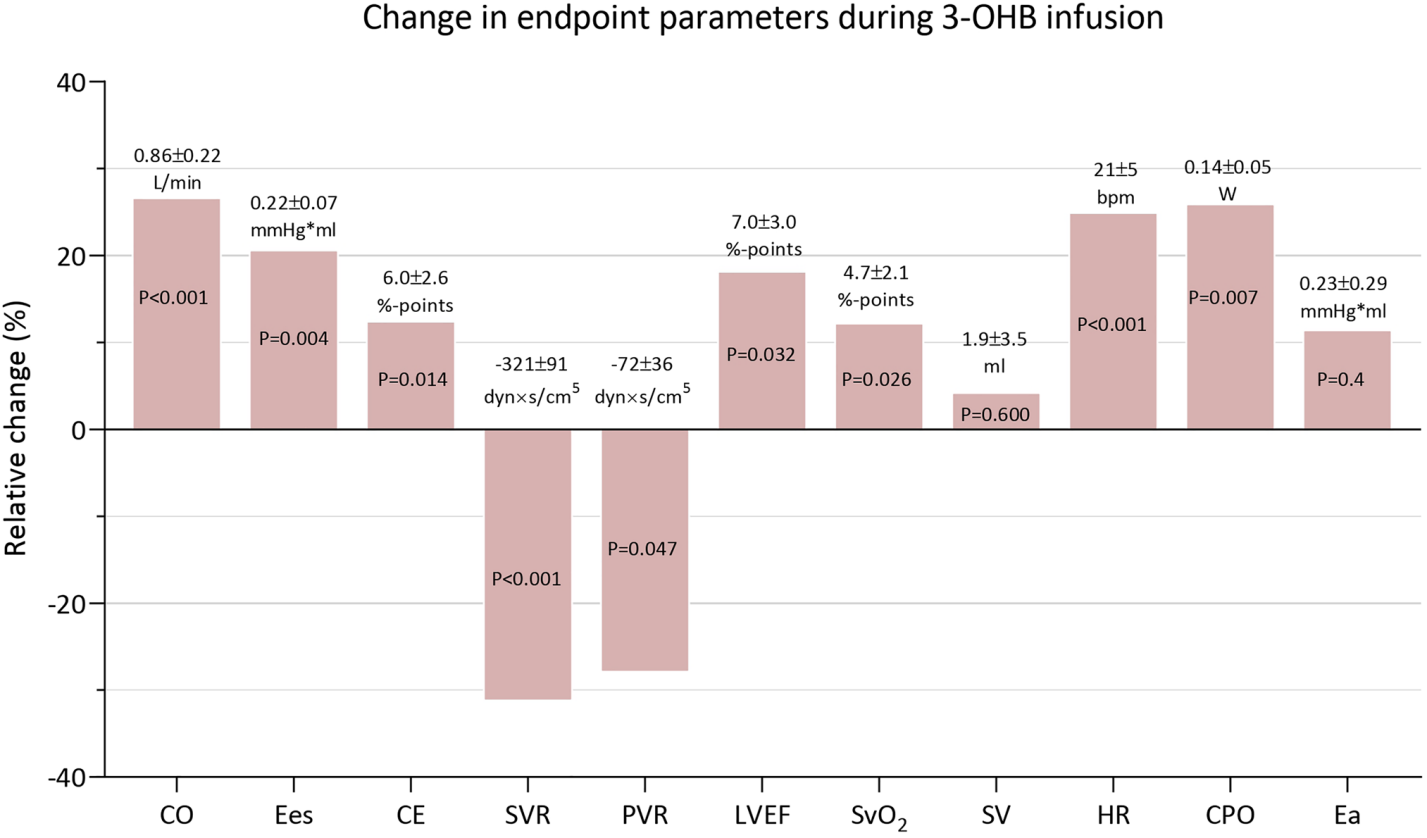
Relative changes in endpoint parameters during 3-OHB infusion Changes in endpoint parameters during 3-OHB infusion versus control through each two-hour intervention period. Y-axis indicates the mean relative change during 3-OHB infusion compared with control infusion. Corresponding mean absolute changes ± standard error of mean (SEM) is listed above or below each bar. *P*-values are derived from the linear mixed model analysis. 16 animals were studied. CO = cardiac output, Ees = end-systolic elastance (the slope of end-systolic pressure-volume relationship; ESPVR), CE = cardiac mechanical efficiency, SVR = systemic vascular resistance, PVR = pulmonary vascular resistance, LVEF = left ventricular ejection fraction, SvO2 = mixed venous oxygen saturation, SV = stroke volume, HR = heart rate, CPO = cardiac power output, Ea = arterial elastance

### Impact of 3-OHB infusion on pressure–volume parameters

Infusion with 3-OHB increased load independent contractility measure Ees by 0.22 mmHg/mL (95% CI 0.08 to 0.37 mmHg/mL, *P* = 0.004; Table 3, Figs. 3 and 4). d*P*/d*t*_max_ also increased. LVEF increased by 7%-points (95% CI 1 to 13%-points, *P* = 0.032). Furthermore, cardiac mechanical efficiency was improved during 3-OHB infusion by 6%-points (95% CI 1 to 11%-points, *P* = 0.014) compared with control. SW, PE, PVA, and CW did not change significantly. Tau was significantly decreased during 3-OHB infusion compared with control. Meanwhile, LVEDV, Ea, and VA-coupling did not differ significantly between treatments. Also, LVESV, LVESP, and LVEDP remained similar during both infusions (Table 3 and Fig. 3).

### Myocardial mitochondrial respiration after 3-OHB infusion

**Fig. 5:**
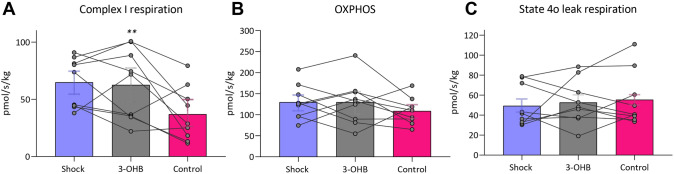
Mitochondrial Function Following 120 Minutes of Treatment. Plots showing the effects of treatment on mitochondrial respiration, oxidative phosphorylation (OXPHOS) capacities and state 4o leak respiration at shock and following 120 min of infusion with 3-OHB and Control. Mitochondrial analysis was performed in nine animals. The boxes display the mean and error bars indicate ± SD. Connected points indicate the repetitive biopsies in each pig. ** indicates *P* < 0.01 for 3-OHB compared with control in the linear mixed model analysis

Out of the 16 pigs included, repeated endomyocardial biopsies were obtained in 9 animals; 5 pigs receiving 3-OHB before control and 4 pigs receiving control before 3-OHB (Supplemental Fig. 4, Supplemental Table 3). Seven pigs did not undergo endomyocardial sampling due to logistical hindrances. Myocardial sampling was safely performed without hemodynamic deterioration (Supplemental Fig. 5). Mitochondrial respiration specific to complex I, which reflects electron transport chain activity associated with the reducing equivalent NADH, was significantly higher during 3-OHB infusion compared with control (Fig. 5, Supplemental Table 2). Meanwhile, OXPHOS capacity, reflecting fully coupled respiration involving complex I and II (including both reducing equivalents NADH and FADH_2_) remained similar between treatments. There was no difference in state 4o leak respiration, which represents non-ATP-generating proton leak across the mitochondrial membrane.

### Impact of 3-OHB treatment on biomarkers

Infusion of 3-OHB was associated with differences in circulating plasma metabolites and biomarkers as FFA concentrations decreased by 0.03 mmol/L (95% CI 0.05 to 0.01 mmol/L, *P* = 0.034; Table 2). Glucose concentrations remained unaltered while lactate concentration was increased by 0.4 mmol/L (0.2 to 0.8 mmol/L, *P* < 0.001) compared with control infusion. pH and bicarbonate levels were increased during 3-OHB infusion compared with control, while the hemoglobin level was increased. Arterial plasma levels of hs-TnI remained similar between treatments (Supplemental Fig. 6).

## Discussion

In this study, we identified the cardiovascular and cardiometabolic effects of infusion with the ketone body 3-OHB in a large-animal model of CS. Our approach allowed for a detailed characterization of cardiac mechanics and metabolic efficiency, offering insights into the underlying myocardial effects of ketone therapy during CS. Utilizing PV loop analysis and high-resolution myocardial respirometry, we provide novel evidence that 3-OHB improves CO and peripheral tissue perfusion in ischemic CS by directly enhancing myocardial contractility by 21% and lowering systemic vascular resistance by 21%, while preserving mitochondrial function. These effects were achieved without altering MAP. Of importance, 3-OHB treatment did not affect PVA or CW, indicating no adverse increase in myocardial oxygen consumption (MvO_2_) [10]. Our approach allowed for a detailed characterization of cardiac mechanics and metabolic efficiency, offering insights into the underlying myocardial effects of ketone therapy during CS. The 3-OHB infusion was well-tolerated, and no ventricular arrhythmias were observed.

### Cardiac output increased during 3-OHB infusion

CS is characterized by myocardial contractile depression and impaired CO, causing hypotension and impaired end-organ perfusion [23, 65]. A mismatch between myocardial oxygen supply and demand further exacerbates cardiac ischemia and challenges cardiac metabolism. Hence, the primary treatment goal in CS is to reestablish organ perfusion by restoring systemic blood pressure and CO [37, 65]. The present study demonstrated that 3-OHB infusion had several beneficial hemodynamic effects in CS. Foremost, the primary endpoint CO improved by 24% compared with control infusion. This improvement in hemodynamic function is similar to low-dose treatment with inotropes and vasopressors such as dobutamine [12], dopamine [15], milrinone [1, 12], and norepinephrine [46]. Additionally, the mortality predictor CPO [14] was improved during 3-OHB infusion. The CO increase was accompanied by several indices of improved peripheral tissue perfusion, including increased SvO_2_, decreased in P(v-a)CO_2_, and increased diuresis, whereas MAP remained unchanged. Thus, treatment with 3-OHB may aid patients with CS by improving cardiac forward flow and end-organ perfusion. Notably, treatment with 3-OHB was associated with increased HR, potentially exacerbating oxygen scarcity for the myocardium [3]. While this may reflect reflex tachycardia due to peripheral vasodilation, PVA and CW, which are correlated to MvO_2_ [58, 59], remained unchanged between the treatments. Also, the administration of 3-OHB was well-tolerated with no adverse ventricular arrhythmias or increase in hs-TnI levels. Our results contrast with a previous study in human patients with CS, in which HR did not change with 3-OHB treatment [5]. However, these patients were already treated with vasoactive drugs, possibly affecting HR and peripheral resistance before 3-OHB administration was initiated while the pigs in the current study were not treated with any vasoactive drugs. Still, the increase in HR might cause some concern and further clinical studies are warranted to investigate the safety of 3-OHB infusion.

### Cardiovascular effects of 3-OHB infusion

Treatment with 3-OHB has been demonstrated to increase CO in patients with CS [5] and heart failure with reduced [4, 44] and preserved LVEF [21]. In these studies, the increase in CO has been attributed to both increased SV and HR [4, 44]. However, it has remained unclear whether these observations are caused by increased contractility, decreased vascular resistance, or both combined. Notably, a recent rodent study reported that 3-OHB increased contractility and caused vasorelaxation in several vascular beds [25], while in healthy pigs, 3-OHB infusion did not change contractility [20]. Intriguingly, both contractility and vascular resistance were altered in our study, demonstrating multifaceted cardiovascular effects of exogenous ketone treatment in ischemic CS which are intricately interconnected within the complex constraints of the cardiovascular system. Also, while cardiac function improved, we observed preserved mitochondrial function indicating no adverse increase in myocardial energy metabolism. Thus, the factors contributing to cardiac enhancement are complex and closely linked. Also noteworthy, a recent ex vivo study in cardiomyocytes from healthy humans and diseased cardiomyocytes from hearts with reduced LVEF, reported that 3-OHB improved contractile function in the diseased cardiomyocytes, but not in the healthy cardiomyocytes [67]. For the first time, the present study demonstrated that 3-OHB infusion significantly increased the load independent contractility measure Ees by 21% in an in vivo large animal model of CS. Furthermore, d*P*/d*t*(max) was significantly increased. Despite increased HR, SV remained relatively unchanged while LVEF increased, suggesting a maintained diastolic function. In fact, the LV diastolic time constant, tau, was significantly decreased during 3-OHB infusion compared with placebo, indicating more efficient diastolic relaxation. While LVEDV and LVEDP were unchanged, this likely reflects the influence of increased HR, as LVEDV and LVEDP are volumetric measurements influenced by preload conditions and HR rather than direct measurements of diastolic function itself. The afterload measurement Ea was not significantly altered during 3-OHB infusion, whereas SVR and PVR decreased during 3-OHB infusion indicative of peripheral and pulmonary vasorelaxation. Unchanged Ea may reflect a decrease in SVR and a compensatory increase in HR [8]. Our findings corroborate with prior research [5, 20, 25] suggesting that 3-OHB enhances myocardial function and reduces vascular resistances, even during CS. This supports the role of 3-OHB in improving CO and end-organ perfusion under severe stress, likely through a combination of direct cardiac and vascular effects.

### Effects of 3-OHB on mechano-energetic and mitochondrial function

The healthy heart is considered a metabolic omnivore with an extensive metabolic flexibility [31]. CS composes a hyperadrenergic state that can decrease metabolic flexibility through insulin resistance and cardiometabolic imbalance [50, 60]. Also, as the heart becomes less efficient due to cardiometabolic imbalance and decreased contractile function, SW is decreased resulting in decreased cardiac mechanical efficiency and ventriculoarterial decoupling [10, 39, 62]. In other words, the heart is working harder but accomplishing less. This puts stress on myocardial mitochondria resulting in a mismatch between energy supply and demand [72]. Notably, cardiac 3-OHB consumption is proportional to circulating levels [40, 41] during heart failure and has demonstrated several beneficial energetic properties as substrate for oxidative metabolism in the failing heart [34]. Also, 3-OHB is readily diffusible to be metabolized through few intermediate steps with a higher ATP per spent oxygen molecule than FFA [71]. 3-OHB has been linked to preserved mitochondrial function [9, 16, 17] and improved recovery of contractile function following ischemia and reperfusion injury [7, 70]. Treatment with 3-OHB may, therefore, have advantageous properties as additional substrate for ATP [71] production during compromised energy supply in CS. Notably, mitochondrial energy substrate shift may occur within the early hours following ischemic injury [54].

In our CS model, 3-OHB infusion resulted in preserved function of myocardial mitochondrial complex I while it was significantly decreased following control infusion. Considering the susceptibility of complex I to ischemic injury [45], these findings suggest a potential mitochondrial-protective effect of 3-OHB during ischemic conditions. These findings are intriguing as we demonstrated improved cardiac systolic and diastolic function accompanied by improved cardiac mechanical efficiency indicating improved LV mechano-energetic function. While the significance of cardiac mechanical efficiency (SW/PVA) is uncertain, related parameters of mechano-energetic function are correlated with mortality in CS [14]. While HR increased significantly, CW remained similar between treatments, suggestive of sustained MvO_2_ despite increased cardiac function.

In animals receiving 3-OHB as the first infusion, complex I activity was preserved at initial shock levels, whereas a decrease was observed in the control animals (Supplemental Fig. 4). In the second infusion period, 3-OHB infusion was associated with an increase in complex I activity with a return to initial shock level respiration. This is the first study to show a myocardial mitochondrial preservation effect of 3-OHB treatment during CS. As an efficient metabolic substrate, 3-OHB enhances mitochondrial energetics by optimizing the redox state, reducing reactive oxygen species (ROS), and improving ATP synthesis efficiency through insulin-independent pathways, thereby optimizing mitochondrial function during insulin-resistance and low energy supply [25, 66]. However, as 3-OHB was not present in the incubator, these effects must have occurred in vivo. This may point to that 3-OHB induces lasting mitochondrial beneficial effects, even when 3-OHB is no longer present.

Indeed, 3-OHB promotes sarcomere shortening and restores contractile function via increased ATP availability, corroborating the enhanced contractility observed in our study and highlighting a direct mitochondrial contribution to myofilament performance [67]. 3-OHB was not present in the incubation chambers during analysis of the permeabilized fibers in the current study. Therefore, the mechanisms of action behind the lasting preservation of mitochondrial function may involve several in vivo metabolic and non-metabolic effects. Indeed, 3-OHB may exert pleiotropic, non-metabolic effects to support mitochondrial preservation. Proposed mechanisms include signaling via the hydroxycarboxylic acid receptor [19, 61], inhibition of histone deacetylases [56], and modulation of transcription factors [22]. These alterations may reduce ROS formation and reinforce mitochondrial antioxidant defense, resulting in lasting protection of myocardial mitochondria [48]. We observed that early 3-OHB infusion (infusion period 1) preserved complex I activity, while later administration (infusion period 2) led to complex I recovery (Supplemental Fig. 4). Similar restorative effects of 3-OHB on mitochondria have been found in skeletal muscle mitochondria in critically ill patients [18]. Conversely, 3-OHB-mediated cardioprotection in CS may involve both metabolic and receptor-mediated mechanisms that stabilize mitochondrial complex I and mitigate mitochondrial injury [54, 66]. Finally, 3-OHB may indirectly preserve mitochondrial function by stabilizing vasomotor tone during intense sympathetic stimulation induced by CS [25]. Notably, when given at reperfusion 3-OHB may be able to reduce infarction size through decreased formation of ROS preservation of mitochondrial DNA integrity and increased autophagy in mice [9]. Still, it remains to be clarified whether the mitochondrial effects of 3-OHB observed in this study represent direct cardioprotective mechanisms through favorable metabolic properties, or if they reflect indirect mitochondrial rescue secondary to improved hemodynamics [54]. The exact underlying mechanisms for the acute mitochondrial changes during 3-OHB treatment were beyond the scope of this study.

Also, it cannot be completely ruled out that the observed increase in complex I activity with 3-OHB, may partly be explained by a substrate shift from FFA to 3-OHB with an improved ATP pr. oxygen ratio as excess FFAs can induce abnormalities in mitochondrial function, including inefficient oxygen usage, excessive mitochondrial metabolism and following increased oxidative damage [72].

In conclusion, this study is the first to comprehensively assess both hemodynamic and cardiac mitochondrial effects of treatment with 3-OHB in a large-animal in vivo model of ischemic CS, utilizing PV loop analysis and high-resolution myocardial respirometry. We unveiled that infusion with 3-OHB enhanced cardiac contractility, preserved mitochondrial function, and optimized hemodynamic performance in CS. These findings provide critical translational insights, bridging the gap between preclinical metabolic studies and clinical hemodynamic observations, highlighting the therapeutic potential of ketone treatment in CS.

### Safety

One pig died from irreversible malignant ventricular arrhythmia during control infusion. No malignant arrhythmias occurred during any intervention infusion for the other animals. Four animals receiving 3-OHB and two animals receiving the control infusion had intermittent supraventricular tachycardias without any apparent hemodynamic significance. Next, in line with other trials, administration of 3-OHB was associated with increased circulating lactate [20, 44]. This is likely caused by substrate competition in which pyruvate is shuttled to lactate [42]. Although, increased circulating lactate can increase CO [27, 29] the lactate levels in the present study remained below a therapeutic significant level and, therefore, was not believed to affect CO or, importantly, represent ischemia or hypoperfusion. Hence, 3-OHB infusion was safely administered and well tolerated.

3-OHB infusion resulted in slight metabolic alkalosis. In the present study, animals were mechanically ventilated they were unable to engage in physiological autoregulation. The increase in pH and bicarbonate could represent a relevant clinical effect, particularly given the metabolic acidosis frequently observed in patients with CS [65].

### Limitations

Several limitations of the present study should be considered. First, findings in animal research may not readily translate into clinical practice. To enhance clinical relevance, we chose human-sized pigs as experimental animals because pigs have close anatomical similarities with the human thoracic anatomy and their cardiovascular and respiratory physiology and biochemical parameters closely resemble those of humans [52]. The study animals were, however, young and healthy, at baseline thus differing from human ischemic CS population. Secondly, CS induced by coronary occlusion through the injection of microspheres into the coronary artery may differ from that resulting from atherosclerotic disease. Notably, arterial levels of lactate do not reflect immediate hypoperfusion and dysregulation in experimental porcine CS through microembolization [47], hence this was not included in the predetermined CS criteria in the present study. Nonetheless, the acute hemodynamic changes of acute CS are expected to be similar.

3-OHB infusion resulted in decreased plasma levels of FFAs. We did not specifically dissect whether reduced circulating FFAs contributed to the hemodynamic benefits of 3-OHB. While excess FFAs may harm mitochondrial function [72] a recent clinical trial used niacin to lower circulating FFAs in patients with heart failure and reduced LVEF. This did not lead to improved hemodynamics compared with placebo [19]. Other investigations examining the reduction of plasma FFAs have reported that lowering these levels does not enhance cardiovascular function [64, 69]. Conversely elevated plasma FFA levels are associated with improved cardiac performance [68]. In a regression analysis, we did not demonstrate a correlation between FFA levels and changes in cardiac performance (Supplemental Material S2). Therefore, it is reasonable to conclude that the observed effects in the current study were most likely due to the administration of 3-OHB. However, the isolated hemodynamic effects of lowering FFAs were not investigated in this study.

We did not measure MvO₂ or direct ATP yield per oxygen for 3-OHB metabolism. Therefore, while our findings suggest improved cardiac function and preserved mitochondrial respiration with 3-OHB, we cannot definitively conclude that 3-OHB is a more oxygen-efficient substrate. While 3-OHB have been shown to be a more oxygen efficient fuel than FFAs [34, 71], future investigations measuring substrate oxidation, MvO₂, and ATP production will be necessary to confirm or refute this potential mechanistic advantage of 3-OHB infusion in CS.

The 3-OHB infusion was hypertonic compared with normal blood tonicity. Infusion of hypertonic fluids is known to cause hemodynamic effects as increased in preload and cardiac contractility as well as reductions in SVR [51]. Therefore, we chose a control infusion with the same tonicity as the 3-OHB infusion. Hence, it is unlikely that hyperosmolality were the cause of the demonstrated hemodynamic effects. Crossover trials inherently carry the risk of carryover effects. We employed a linear mixed model that adjusted for the interaction of period and carryover effects. While some interactions were identified regarding secondary endpoints our analysis revealed overall no significant impact on the primary endpoint.

Finally, though the use of permeabilized fibers to assess mitochondrial respiratory capacity is a well-validated method for investigating mitochondrial function following ischemia [13], the in-situ preparation does not fully replicate the physiological milieu [6]. Consequently, it remains uncertain whether our findings can be directly extrapolated to the whole organ level. While high-resolution respirometry offers significant insights into mitochondrial function within a controlled extracellular environment, it does not allow for the evaluation of cellular energetics in intact tissue. Also, as we aimed to investigate the lasting effect of 3-OHB treatment, 3-OHB was not present in the incubation chambers during analysis. Hence, further studies are warranted, to evaluate the direct oxidative capacity of 3-OHB within the mitochondria in CS.

## Conclusions

Infusion of 3-OHB increased cardiac output and peripheral perfusion during cardiogenic shock compared with euvolemic, equimolar hypertonic saline control. The cardiac output increase was mediated through increased left ventricular systolic and diastolic function and increased heart rate accompanied by decreased vascular resistance without affecting blood pressure. These effects were associated with improved myocardial mechano-energetic and mitochondrial function. Therefore, 3-OHB could be a promising treatment in cardiogenic shock.

## Supplementary Information

Below is the link to the electronic supplementary material.

Supplementary file1 (DOCX 6517 KB)

## Data Availability

The datasets used and analysed during the current study are available from the corresponding author on reasonable request.

## Abbreviations

3-OHB: 3-Hydroxybutyrate
CI: Confidence interval
CO: Cardiac output
CPO: Cardiac power output
CS: Cardiogenic shock
CW: Cardiac work
dP/dt(max): Maximum rate of pressure increase
Ea: Arterial elastance
Ees: End-systolic elastance
ESPVR: End-systolic pressure–volume relationship
FFA: Free fatty acids
HR: Heart rate
hs-TnI: High-sensitivity troponin I
IQR: Interquartile range
LV: Left ventricle
LVEDP: Left ventricular end-diastolic pressure
LVEDV: Left ventricular end-diastolic volume
LVEF: Left ventricular ejection fraction
LVESV: Left ventricular end-systolic volume
LVESP: Left ventricular end-systolic pressure
MAP: Mean arterial pressure
mPAP: Mean pulmonary artery pressure
OXPHOS: Oxidative phosphorylation
PA: Pulmonary artery
PAWP: Pulmonary artery wedge pressure
PaPi: Pulmonary artery pulsatility index
PE: Potential energy
P(v-a)CO_2_: Veno-arterial CO_2_ difference
PVA: Pressure–volume area
PV: Pressure–volume
PVR: Pulmonary vascular resistance
RAP: Right atrial pressure
SD: Standard deviation
SEM: Standard error of mean
SvO_2_: Mixed venous oxygen saturation
SV: Stroke volume
SVR: Systemic vascular resistance
SW: Stroke work
Tau: LV diastolic time constant
VA: Ventriculo-arterial

## References

[1] Anderson JL, Baim DS, Fein SA, Goldstein RA, LeJemtel TH, Likoff MJ (1987) Efficacy and safety of sustained (48 hour) intravenous infusions of milrinone in patients with severe congestive heart failure: a multicenter study. J Am Coll Cardiol 9:711–722. 10.1016/S0735-1097(87)80223-1 3549837 10.1016/s0735-1097(87)80223-1

[2] Annoni F, Su F, Peluso L, Lisi I, Caruso E, Pischiutta F, Gouvea Bogossian E, Garcia B, Njimi H, Vincent J-L, Gaspard N, Ferlini L, Creteur J, Zanier ER, Taccone FS (2024) Infusion of sodium DL-3-ß-hydroxybutyrate decreases cerebral injury biomarkers after resuscitation in experimental cardiac arrest. Crit Care 28:314. 10.1186/s13054-024-05106-8 39304944 10.1186/s13054-024-05106-8 PMC11414246

[3] Antonopoulos AS, Goliopoulou A, Vogiatzi G, Tousoulis D (2018) Myocardial oxygen consumption. Coronary artery disease. Elsevier, Amsterdam, pp 127–136

[4] Berg-Hansen K, Gopalasingam N, Christensen KH, Ladefoged B, Andersen MJ, Poulsen SH, Borlaug BA, Nielsen R, Møller N, Wiggers H (2024) Cardiovascular Effects of Oral Ketone Ester Treatment in Patients With Heart Failure With Reduced Ejection Fraction: A Randomized, Controlled, Double-Blind Trial. Circulation. 10.1161/CIRCULATIONAHA.123.067971 10.1161/CIRCULATIONAHA.123.067971 PMC11081479 38533643

[5] Berg-Hansen K, Christensen KH, Gopalasingam N, Nielsen R, Eiskjær H, Møller N, Birkelund T, Christensen S, Wiggers H (2023) Beneficial effects of ketone ester in patients with cardiogenic shock: a randomized, controlled, double-blind trial. JACC Heart Fail 11:1337–1347. 10.1016/j.jchf.2023.05.029 37452805 10.1016/j.jchf.2023.05.029

[6] Brand MD, Nicholls DG (2011) Assessing mitochondrial dysfunction in cells. Biochemical Journal 435:297–312. 10.1042/BJ20110162 21726199 10.1042/BJ20110162 PMC3076726

[7] Byrne NJ, Soni S, Takahara S, Ferdaoussi M, Al Batran R, Darwesh AM, Levasseur JL, Beker D, Vos DY, Schmidt MA, Alam AS, Maayah ZH, Schertzer JD, Seubert JM, Ussher JR, Dyck JRB (2020) Chronically Elevating Circulating Ketones Can Reduce Cardiac Inflammation and Blunt the Development of Heart Failure. Circ Heart Fail 13. 10.1161/CIRCHEARTFAILURE.119.006573 10.1161/CIRCHEARTFAILURE.119.006573 32493060

[8] Chirinos JA, Rietzschel ER, Shiva-Kumar P, De Buyzere ML, Zamani P, Claessens T, Geraci S, Konda P, De Bacquer D, Akers SR, Gillebert TC, Segers P (2014) Effective Arterial Elastance Is Insensitive to Pulsatile Arterial Load. Hypertension 64:1022–1031. 10.1161/HYPERTENSIONAHA.114.03696 25069668 10.1161/HYPERTENSIONAHA.114.03696

[9] Chu Y, Hua Y, He L, He J, Chen Y, Yang J, Mahmoud I, Zeng F, Zeng X, Benavides GA, Darley-Usmar VM, Young ME, Ballinger SW, Prabhu SD, Zhang C, Xie M (2024) β-hydroxybutyrate administered at reperfusion reduces infarct size and preserves cardiac function by improving mitochondrial function through autophagy in male mice. J Mol Cell Cardiol 186:31–44. 10.1016/j.yjmcc.2023.11.001 37979443 10.1016/j.yjmcc.2023.11.001 PMC11094739

[10] Curran J, Burkhoff D, Kloner RA (2019) Beyond Reperfusion: Acute Ventricular Unloading and Cardioprotection During Myocardial Infarction. J Cardiovasc Transl Res 12:95–106. 10.1007/s12265-019-9863-z 30671717 10.1007/s12265-019-9863-z PMC6497619

[11] Doerrier C, Garcia-Souza LF, Krumschnabel G, Wohlfarter Y, Mészáros AT, Gnaiger E (2018) High-Resolution FluoRespirometry and OXPHOS Protocols for Human Cells, Permeabilized Fibers from Small Biopsies of Muscle, and Isolated Mitochondria. pp 31–70 10.1007/978-1-4939-7831-1_3 29850993

[12] Eichhorn EJ, Konstam MA, Weiland DS, Roberts DJ, Martin TT, Stransky NB, Salem DN (1987) Differential effects of milrinone and dobutamine on right ventricular preload, afterload and systolic performance in congestive heart failure secondary to ischemic or idiopathic dilated cardiomyopathy. Am J Cardiol 60:1329–1333. 10.1016/0002-9149(87)90616-3 3687783 10.1016/0002-9149(87)90616-3

[13] Eickelmann C, Lieder HR, Shehada S-E, Thielmann M, Heusch G, Kleinbongard P (2023) Mitochondrial respiration analysis in permeabilized porcine left ventricular and human right atrial specimens with ischemia-reperfusion. American Journal of Physiology-Heart and Circulatory Physiology 325:H125–H135. 10.1152/ajpheart.00172.2023 37235522 10.1152/ajpheart.00172.2023

[14] Fincke R, Hochman JS, Lowe AM, Menon V, Slater JN, Webb JG, LeJemtel TH, Cotter G, Investigators SHOCK (2004) Cardiac power is the strongest hemodynamic correlate of mortality in cardiogenic shock: a report from the SHOCK trial registry. J Am Coll Cardiol 44:340–348. 10.1016/j.jacc.2004.03.060 15261929 10.1016/j.jacc.2004.03.060

[15] Francis GS, Sharma B, Hodges M (1982) Comparative hemodynamic effects of dopamine and dobutamine in patients with acute cardiogenic circulatory collapse. Am Heart J 103:995–1000. 10.1016/0002-8703(82)90562-2 7081040 10.1016/0002-8703(82)90562-2

[16] Frey S, Geffroy G, Desquiret-Dumas V, Gueguen N, Bris C, Belal S, Amati-Bonneau P, Chevrollier A, Barth M, Henrion D, Lenaers G, Bonneau D, Reynier P, Procaccio V (2017) The addition of ketone bodies alleviates mitochondrial dysfunction by restoring complex I assembly in a MELAS cellular model. Biochim Biophys Acta (BBA) Mol Basis Dis 1863:284–291. 10.1016/j.bbadis.2016.10.028 10.1016/j.bbadis.2016.10.028 27815040

[17] Gambardella J, Jankauskas SS, Kansakar U, Varzideh F, Avvisato R, Prevete N, Sidoli S, Mone P, Wang X, Lombardi A, Santulli G (2023) Ketone bodies rescue mitochondrial dysfunction via epigenetic remodeling. JACC Basic Transl Sci 8:1123–1137. 10.1016/j.jacbts.2023.03.014 37791311 10.1016/j.jacbts.2023.03.014 PMC10543927

[18] Genserová L, Duška F, Krajčová A (2024) β-hydroxybutyrate exposure restores mitochondrial function in skeletal muscle satellite cells of critically ill patients. Clin Nutr 43:1250–1260. 10.1016/j.clnu.2024.04.009 38653008 10.1016/j.clnu.2024.04.009

[19] Gopalasingam N, Christensen KH, Berg Hansen K, Nielsen R, Johannsen M, Gormsen LC, Boedtkjer E, Nørregaard R, Møller N, Wiggers H (2023) Stimulation of the hydroxycarboxylic acid receptor 2 with the ketone body 3-hydroxybutyrate and niacin in patients with chronic heart failure: hemodynamic and metabolic effects. J Am Heart Assoc 12:e029849. 10.1161/JAHA.123.029849 37301762 10.1161/JAHA.123.029849 PMC10356045

[20] Gopalasingam N, Moeslund N, Christensen KH, Berg-Hansen K, Seefeldt J, Homilius C, Nielsen EN, Dollerup MR, Alstrup Olsen AK, Johannsen M, Boedtkjer E, Møller N, Eiskjær H, Gormsen LC, Nielsen R, Wiggers H (2024) Enantiomer-specific cardiovascular effects of the ketone body 3-hydroxybutyrate. J Am Heart Assoc. 10.1161/JAHA.123.033628 38563382 10.1161/JAHA.123.033628 PMC11262493

[21] Gopalasingam N, Berg-Hansen K, Christensen KH, Ladefoged BT, Poulsen SH, Andersen MJ, Borlaug B, Nielsen R, Møller N, Wiggers H (2024) Randomized crossover trial of 2-week ketone ester treatment in patients with Type 2 diabetes and heart failure with preserved ejection fraction. Circulation. 10.1161/CIRCULATIONAHA.124.069732 39162035 10.1161/CIRCULATIONAHA.124.069732

[22] Han Y, Bedarida T, Ding Y, Somba BK, Lu Q, Wang Q, Song P, Zou M-H (2018) β-Hydroxybutyrate prevents vascular senescence through hnRNP A1-mediated upregulation of Oct4. Mol Cell 71:1064-1078.e5. 10.1016/j.molcel.2018.07.036 30197300 10.1016/j.molcel.2018.07.036 PMC6230553

[23] Harjola V-P, Lassus J, Sionis A, Køber L, Tarvasmäki T, Spinar J, Parissis J, Banaszewski M, Silva-Cardoso J, Carubelli V, Di Somma S, Tolppanen H, Zeymer U, Thiele H, Nieminen MS, Mebazaa A, CardShock Study Investigators, GREAT network (2015) Clinical picture and risk prediction of short-term mortality in cardiogenic shock. Eur J Heart Fail 17:501–509. 10.1002/ejhf.260 25820680 10.1002/ejhf.260

[24] Hevrøy O, Reikerås O, Grundnes O, Mjøs OD (1988) Cardiovascular effects of positive end-expiratory pressure during acute left ventricular failure in dogs. Clin Physiol 8:287–301. 10.1111/j.1475-097x.1988.tb00271.x 3042273 10.1111/j.1475-097x.1988.tb00271.x

[25] Homilius C, Seefeldt JM, Axelsen JS, Pedersen TM, Sørensen TM, Nielsen R, Wiggers H, Hansen J, Matchkov VV, Bøtker HE, Boedtkjer E (2023) Ketone body 3-hydroxybutyrate elevates cardiac output through peripheral vasorelaxation and enhanced cardiac contractility. Basic Res Cardiol 118:37. 10.1007/s00395-023-01008-y 37688627 10.1007/s00395-023-01008-y PMC10492777

[26] Hørsdal OK (2025) Can utilization of the venous-to-arterial carbon dioxide difference improve patient outcomes in cardiogenic shock? A narrative review. Am Heart J Plus Cardiol Res Pract 50:100504. 10.1016/j.ahjo.2025.100504 10.1016/j.ahjo.2025.100504 PMC11840508 39981412

[27] Hørsdal OK, Moeslund N, Berg-Hansen K, Nielsen R, Møller N, Eiskjær H, Wiggers H, Gopalasingam N (2024) Lactate infusion elevates cardiac output through increased heart rate and decreased vascular resistance: a randomised, blinded, crossover trial in a healthy porcine model. J Transl Med 22:285. 10.1186/s12967-024-05064-3 38493167 10.1186/s12967-024-05064-3 PMC10943846

[28] Hørsdal OK, Wethelund KL, Gopalasingam N, Lyhne MD, Ellegaard MS, Møller-Helgestad OK, Ravn HB, Wiggers H, Christensen S, Berg-Hansen K (2024) Cardiovascular effects of increasing positive end-expiratory pressure in a model of left ventricular cardiogenic shock in female pigs. Anesthesiology. 10.1097/ALN.0000000000005201 39186681 10.1097/ALN.0000000000005201

[29] Hørsdal OK, Ellegaard MS, Larsen AM, Guldbrandsen H, Moeslund N, Møller JE, Helgestad OKL, Ravn HB, Wiggers H, Nielsen R, Gopalasingam N, Berg-Hansen K (2025) Lactate infusion improves cardiac function in a porcine model of ischemic cardiogenic shock. Crit Care 29:113. 10.1186/s13054-025-05346-2 40083003 10.1186/s13054-025-05346-2 PMC11907994

[30] Kleinbongard P, Heusch G (2022) A fresh look at coronary microembolization. Nat Rev Cardiol 19:265–280. 10.1038/s41569-021-00632-2 34785770 10.1038/s41569-021-00632-2 PMC8593642

[31] Kolwicz SC, Purohit S, Tian R (2013) Cardiac metabolism and its interactions with contraction, growth, and survival of cardiomyocytes. Circ Res 113:603–616. 10.1161/CIRCRESAHA.113.302095 23948585 10.1161/CIRCRESAHA.113.302095 PMC3845521

[32] Kuznetsov AV, Veksler V, Gellerich FN, Saks V, Margreiter R, Kunz WS (2008) Analysis of mitochondrial function in situ in permeabilized muscle fibers, tissues and cells. Nat Protoc 3:965–976. 10.1038/nprot.2008.61 18536644 10.1038/nprot.2008.61

[33] Lim HS, Gustafsson F (2020) Pulmonary artery pulsatility index: physiological basis and clinical application. Eur J Heart Fail 22:32–38. 10.1002/ejhf.1679 31782244 10.1002/ejhf.1679

[34] Lopaschuk GD, Dyck JRB (2023) Ketones and the cardiovascular system. Nat Cardiovasc Res 2:425–437. 10.1038/s44161-023-00259-1 39196044 10.1038/s44161-023-00259-1

[35] Lopaschuk GD, Karwi QG, Tian R, Wende AR, Abel ED (2021) Cardiac energy metabolism in heart failure. Circ Res 128:1487–1513. 10.1161/CIRCRESAHA.121.318241 33983836 10.1161/CIRCRESAHA.121.318241 PMC8136750

[36] Ltaief Z, Schneider AG, Liaudet L (2021) Pathophysiology and clinical implications of the veno-arterial PCO2 gap. Crit Care 25:318. 10.1186/s13054-021-03671-w 34461974 10.1186/s13054-021-03671-w PMC8407023

[37] Møller JE, Engstrøm T, Jensen LO, Eiskjær H, Mangner N, Polzin A, Schulze PC, Skurk C, Nordbeck P, Clemmensen P, Panoulas V, Zimmer S, Schäfer A, Werner N, Frydland M, Holmvang L, Kjærgaard J, Sørensen R, Lønborg J, Lindholm MG, Udesen NLJ, Junker A, Schmidt H, Terkelsen CJ, Christensen S, Christiansen EH, Linke A, Woitek FJ, Westenfeld R, Möbius-Winkler S, Wachtell K, Ravn HB, Lassen JF, Boesgaard S, Gerke O, Hassager C (2024) Microaxial flow pump or standard care in infarct-related cardiogenic shock. N Engl J Med 390:1382–1393. 10.1056/NEJMoa2312572 38587239 10.1056/NEJMoa2312572

[38] Møller-Helgestad OK, Ravn HB, Møller JE (2018) Large porcine model of profound acute ischemic cardiogenic shock. Methods Mol Biol 1816:343–352. 10.1007/978-1-4939-8597-5_27 29987833 10.1007/978-1-4939-8597-5_27

[39] Monge García MI, Santos A (2020) Understanding ventriculo-arterial coupling. Ann Transl Med 8:795. 10.21037/atm.2020.04.10 32647720 10.21037/atm.2020.04.10 PMC7333110

[40] Monzo L, Sedlacek K, Hromanikova K, Tomanova L, Borlaug BA, Jabor A, Kautzner J, Melenovsky V (2021) Myocardial ketone body utilization in patients with heart failure: the impact of oral ketone ester. Metabolism 115:154452. 10.1016/j.metabol.2020.154452 33248064 10.1016/j.metabol.2020.154452

[41] Murashige D, Jang C, Neinast M, Edwards JJ, Cowan A, Hyman MC, Rabinowitz JD, Frankel DS, Arany Z (2020) Comprehensive quantification of fuel use by the failing and nonfailing human heart. Science 370:364–368. 10.1126/science.abc8861 33060364 10.1126/science.abc8861 PMC7871704

[42] Ng SM, Neubauer S, Rider OJ (2023) Myocardial metabolism in heart failure. Curr Heart Fail Rep 20:63–75. 10.1007/s11897-023-00589-y 36800045 10.1007/s11897-023-00589-y PMC9977885

[43] Ng YH, Koay YC, Marques FZ, Kaye DM, O’Sullivan JF (2024) Leveraging metabolism for better outcomes in heart failure. Cardiovasc Res. 10.1093/cvr/cvae216 39351766 10.1093/cvr/cvae216 PMC11630082

[44] Nielsen R, Møller N, Gormsen LC, Tolbod LP, Hansson NH, Sorensen J, Harms HJ, Frøkiær J, Eiskjaer H, Jespersen NR, Mellemkjaer S, Lassen TR, Pryds K, Bøtker HE, Wiggers H (2019) Cardiovascular effects of treatment with the ketone body 3-hydroxybutyrate in chronic heart failure patients. Circulation 139:2129–2141. 10.1161/CIRCULATIONAHA.118.036459 30884964 10.1161/CIRCULATIONAHA.118.036459 PMC6493702

[45] Paradies G, Petrosillo G, Pistolese M, Di Venosa N, Federici A, Ruggiero FM (2004) Decrease in mitochondrial complex I activity in ischemic/reperfused rat heart. Circ Res 94:53–59. 10.1161/01.RES.0000109416.56608.64 14656928 10.1161/01.RES.0000109416.56608.64

[46] Perez P, Kimmoun A, Blime V, Levy B (2014) Increasing mean arterial pressure in cardiogenic shock secondary to myocardial infarction. Shock 41:269–274. 10.1097/SHK.0000000000000099 24509521 10.1097/SHK.0000000000000099

[47] Riehle C, Sieweke J-T, Udesen NLJ, Helgestad OKL, Froese N, Ravn HB, Lichtinghagen R, Møller JE, Bauersachs J, Schäfer A (2024) Circulating biomarkers of the CS4P and CLIP scores are not altered in a pig model of acute cardiogenic shock and additional short-term circulatory support. Int J Cardiol 401:131699. 10.1016/j.ijcard.2023.131699 38182061 10.1016/j.ijcard.2023.131699

[48] Rojas-Morales P, Pedraza-Chaverri J, Tapia E (2020) Ketone bodies, stress response, and redox homeostasis. Redox Biol 29:101395. 10.1016/j.redox.2019.101395 31926621 10.1016/j.redox.2019.101395 PMC6911969

[49] Sagawa K (1981) The end-systolic pressure-volume relation of the ventricle: definition, modifications and clinical use. Circulation 63:1223–1227. 10.1161/01.cir.63.6.1223 7014027 10.1161/01.cir.63.6.1223

[50] Scheen M, Giraud R, Bendjelid K (2021) Stress hyperglycemia, cardiac glucotoxicity, and critically ill patient outcomes current clinical and pathophysiological evidence. Physiol Rep. 10.14814/phy2.14713 33463901 10.14814/phy2.14713 PMC7814494

[51] Schroth M, Plank C, Meiβner U, Eberle K-P, Weyand M, Cesnjevar R, Dötsch J, Rascher W (2006) Hypertonic-hyperoncotic solutions improve cardiac function in children after open-heart surgery. Pediatrics 118:e76–e84. 10.1542/peds.2005-2795 16751617 10.1542/peds.2005-2795

[52] Schüttler D, Tomsits P, Bleyer C, Vlcek J, Pauly V, Hesse N, Sinner M, Merkus D, Hamers J, Kääb S, Clauss S (2022) A practical guide to setting up pig models for cardiovascular catheterization, electrophysiological assessment and heart disease research. Lab Anim (NY) 51:46–67. 10.1038/s41684-021-00909-6 35087256 10.1038/s41684-021-00909-6

[53] Seefeldt JM, Lassen TR, Hjortbak MV, Jespersen NR, Kvist F, Hansen J, Bøtker HE (2021) Cardioprotective effects of empagliflozin after ischemia and reperfusion in rats. Sci Rep 11:9544. 10.1038/s41598-021-89149-9 33953281 10.1038/s41598-021-89149-9 PMC8100147

[54] Seefeldt JM, Libai Y, Berg K, Jespersen NR, Lassen TR, Dalsgaard FF, Ryhammer P, Pedersen M, Ilkjaer LB, Hu MA, Erasmus ME, Nielsen RR, Bøtker HE, Caspi O, Eiskjær H, Moeslund N (2024) Effects of ketone body 3-hydroxybutyrate on cardiac and mitochondrial function during donation after circulatory death heart transplantation. Sci Rep 14:757. 10.1038/s41598-024-51387-y 38191915 10.1038/s41598-024-51387-y PMC10774377

[55] Senzaki H, Chen CH, Kass DA (1996) Single-beat estimation of end-systolic pressure-volume relation in humans. A new method with the potential for noninvasive application. Circulation 94:2497–2506. 10.1161/01.cir.94.10.2497 8921794 10.1161/01.cir.94.10.2497

[56] Shimazu T, Hirschey MD, Newman J, He W, Shirakawa K, Le Moan N, Grueter CA, Lim H, Saunders LR, Stevens RD, Newgard CB, Farese RV, de Cabo R, Ulrich S, Akassoglou K, Verdin E (2013) Suppression of oxidative stress by β-hydroxybutyrate, an endogenous histone deacetylase inhibitor. Science (1979) 339:211–214. 10.1126/science.1227166 10.1126/science.1227166 PMC3735349 23223453

[57] Smiseth OA, Mjøs OD (1982) A reproducible and stable model of acute ischaemic left ventricular failure in dogs. Clin Physiol 2:225–239. 10.1111/j.1475-097x.1982.tb00027.x 6889941 10.1111/j.1475-097x.1982.tb00027.x

[58] Suga H (1979) Total mechanical energy of a ventricle model and cardiac oxygen consumption. Am J Physiol 236:H498-505. 10.1152/ajpheart.1979.236.3.H498 426086 10.1152/ajpheart.1979.236.3.H498

[59] Suga H, Igarashi Y, Yamada O, Goto Y (1986) Cardiac oxygen consumption and systolic pressure volume area. Basic Res Cardiol 81(Suppl 1):39–50. 10.1007/978-3-662-11374-5_5 3790043 10.1007/978-3-662-11374-5_5

[60] Sun Q, Karwi QG, Wong N, Lopaschuk GD (2024) Advances in myocardial energy metabolism: metabolic remodelling in heart failure and beyond. Cardiovasc Res. 10.1093/cvr/cvae231 39453987 10.1093/cvr/cvae231 PMC11646102

[61] Taggart AKP, Kero J, Gan X, Cai T-Q, Cheng K, Ippolito M, Ren N, Kaplan R, Wu K, Wu T-J, Jin L, Liaw C, Chen R, Richman J, Connolly D, Offermanns S, Wright SD, Waters MG (2005) (d)-β-hydroxybutyrate inhibits adipocyte lipolysis via the nicotinic acid receptor PUMA-G. J Biol Chem 280:26649–26652. 10.1074/jbc.C500213200 15929991 10.1074/jbc.C500213200

[62] Tehrani BN, Truesdell AG, Psotka MA, Rosner C, Singh R, Sinha SS, Damluji AA, Batchelor WB (2020) A standardized and comprehensive approach to the management of cardiogenic shock. JACC Heart Fail 8:879–891. 10.1016/j.jchf.2020.09.005 33121700 10.1016/j.jchf.2020.09.005 PMC8167900

[63] Thiele H, Ohman EM, Desch S, Eitel I, de Waha S (2015) Management of cardiogenic shock. Eur Heart J 36:1223–1230. 10.1093/eurheartj/ehv051 25732762 10.1093/eurheartj/ehv051

[64] Tuunanen H, Engblom E, Naum A, Någren K, Hesse B, Airaksinen KEJ, Nuutila P, Iozzo P, Ukkonen H, Opie LH, Knuuti J (2006) Free fatty acid depletion acutely decreases cardiac work and efficiency in cardiomyopathic heart failure. Circulation 114:2130–2137. 10.1161/CIRCULATIONAHA.106.645184 17088453 10.1161/CIRCULATIONAHA.106.645184

[65] van Diepen S, Katz JN, Albert NM, Henry TD, Jacobs AK, Kapur NK, Kilic A, Menon V, Ohman EM, Sweitzer NK, Thiele H, Washam JB, Cohen MG (2017) Contemporary Management of Cardiogenic Shock: A Scientific Statement From the American Heart Association. Circulation 136. 10.1161/CIR.0000000000000525 10.1161/CIR.0000000000000525 28923988

[66] Veech RL (2004) The therapeutic implications of ketone bodies: the effects of ketone bodies in pathological conditions: ketosis, ketogenic diet, redox states, insulin resistance, and mitochondrial metabolism. Prostaglandins Leukot Essent Fatty Acids 70:309–319. 10.1016/j.plefa.2003.09.007 14769489 10.1016/j.plefa.2003.09.007

[67] Vite A, Matsuura TR, Bedi KC, Flam EL, Arany Z, Kelly DP, Margulies KB (2024) Functional impact of alternative metabolic substrates in failing human cardiomyocytes. JACC Basic Transl Sci 9:1–15. 10.1016/j.jacbts.2023.07.009 38362346 10.1016/j.jacbts.2023.07.009 PMC10864907

[68] Watson WD, Green PG, Lewis AJM, Arvidsson P, De Maria GL, Arheden H, Heiberg E, Clarke WT, Rodgers CT, Valkovič L, Neubauer S, Herring N, Rider OJ (2023) Retained metabolic flexibility of the failing human heart. Circulation 148:109–123. 10.1161/CIRCULATIONAHA.122.062166 37199155 10.1161/CIRCULATIONAHA.122.062166 PMC10417210

[69] Wiggers H, Nørrelund H, Nielsen SS, Andersen NH, Nielsen-Kudsk JE, Christiansen JS, Nielsen TT, Møller N, Bøtker HE (2005) Influence of insulin and free fatty acids on contractile function in patients with chronically stunned and hibernating myocardium. Am J Physiol-Heart Circ Physiol 289:H938–H946. 10.1152/ajpheart.00150.2005 15805229 10.1152/ajpheart.00150.2005

[70] Yurista SR, Matsuura TR, Silljé HHW, Nijholt KT, McDaid KS, Shewale SV, Leone TC, Newman JC, Verdin E, van Veldhuisen DJ, de Boer RA, Kelly DP, Westenbrink BD (2021) Ketone ester treatment improves cardiac function and reduces pathologic remodeling in preclinical models of heart failure. Circ Heart Fail. 10.1161/CIRCHEARTFAILURE.120.007684 33356362 10.1161/CIRCHEARTFAILURE.120.007684 PMC7819534

[71] Yurista SR, Eder RA, Welsh A, Jiang W, Chen S, Foster AN, Mauskapf A, Tang WHW, Hucker WJ, Coll-Font J, Rosenzweig A, Nguyen CT (2023) Ketone ester supplementation suppresses cardiac inflammation and improves cardiac energetics in a swine model of acute myocardial infarction. Metabolism 145:155608. 10.1016/j.metabol.2023.155608 37268056 10.1016/j.metabol.2023.155608 PMC10330653

[72] Zhou B, Tian R (2018) Mitochondrial dysfunction in pathophysiology of heart failure. J Clin Investig 128:3716–3726. 10.1172/JCI120849 30124471 10.1172/JCI120849 PMC6118589

