# Supplementary material for: The ketone body 3-hydroxybutyrate increases cardiac output and cardiac contractility in a porcine model of cardiogenic shock: a randomized, blinded, crossover trial
Source: Basic Res Cardiol. 2025 Apr 12;120(3):579–96. doi: 10.1007/s00395-025-01103-2 (PMC12159096; doi:10.1007/s00395-025-01103-2)
Supplement: Supplementary file 1 — Supplementary file1 (DOCX 6517 KB) [file 395_2025_1103_MOESM1_ESM.docx]

**Supplemental Material S1:** Detailed Protocol on Endomyocardial Mitochondrial Analysis

Mitochondrial respiratory capacity was measured using high resolution respirometry (*Oxygraph 2K, Oroboros Instruments, Innsbruck, Austria)*. Briefly, a flexible biopsy forceps (*Jawz^TM^, Argon Medical Devices, USA)* was advanced through the left carotid artery to the apex of the LV under fluoroscopic guidance. Endomyocardial biopsies were immediately transferred to an ice-cold biopsy preserving solution (BIOPS: 10 Ca-EGTA buffer, 10-4 free Ca2+, 20 imidazole, 20 taurine, 50 K-MES, 0.5 dithiothreitol, 6.56 MgCl2, 5.77 ATP, and 15 phosphocreatine, pH 7.1). Fiber bundles (~1.5mg) were prepared by manual dissection and permeabilized in cold BIOPS mixed with saponin (50 μg mL^-1^) for 30 min. Following permeabilizations, the fiber bundles were rinsed twice (10x2 min.) in a cold mitochondrial respiration medium MiR05 (in mmol L-1: 110 sucrose, 60 K-lactobionate, 0.5 EGTA, 0,1% BSA, 3 mgCl2, 20 taurine, 10 KH2PO4 and 20 Hepes; pH 7.1) and added to the chambers of the Oxygraph-2K. The following substrate protocol was used to evaluate mitochondrial respiration: Glutamate (10 mmol/L) and Malate (2 mmol/L). Subsequent addition of ADP (5 mmol/L) allows complex I mediated respiration with electron flow through the mitochondrial ATPase. Succinate (10 mmol/L) was added to stimulate maximal respiration with electron flow through complex I+II. Oligomycin (complex V inhibitor) (2 μg/mL) was added to evaluate state 4o leak respiration. Final addition of rotenone (complex I inhibitor) (0.5 μmol/L) and antimycin A (complex III inhibitor) (2.5 mmol/l) allows measurement of residual oxygen consumption. To avoid any O2 limitations to respiration the chambers were hyperoxygenated and all measurements were carried out in duplicate. The integrity of the outer mitochondrial membrane was tested by the addition of cytochrome c (10 μmol/L). An increase of >10% in the oxygen consumption rate led to exclusion. The data was corrected for residual oxygen consumption and the OXPHOS capacity was calculated as respiration for complex I+II subtracted with leak state respiration.

**Supplemental Material S2:** Regression Analysis of 3-OHB levels, FFA levels and Cardiac Output

**Statistical Analysis**
We first performed an overall multiple linear regression analysis with change in cardiac output as the dependent variable and 3-OHB and FFA as independent predictors.

To investigate potential differences between the two treatment periods, we stratified the data by period and performed separate regression analyses for period 1 and period 2 using the same predictor variables. Finally, we tested for effect modification by treatment period by including interaction terms between period and each of the predictors in an interaction model.

**Results**
In the overall regression model, higher 3-OHB levels were significantly associated with increases in cardiac output (P<0.001), while FFA levels were not significantly associated (P=0.58). Stratified analyses revealed similar patterns in both periods:

- In period 1, 3-OHB was significant (P=0.002) with no significant effect of FFA (P=0.235)
- In period 2, 3-OHB with significant (P=0.043), with no significant effect of FFA (P=0.889).

**Conclusion**
These findings indicate that FFA levels do not significantly influence cardiac output during 3-OHB infusion.

**Supplemental Figure 1:** Stabile Cardiac Dysfunction for 180 minutes of CS in Animals Receiving Control

Cardiac output and mixed venous oxygen saturation (SvO_2_) in healthy state, right after onset of cardiogenic shock (CS) following embolization, after 60 minutes of no-touch (Shock) and for 180 minutes in animals receiving control infusion in the first intervention period (n=8). Black line is mean ± SEM. Each animal is displayed as pink lines.

**Supplemental Figure 2:** Mean Hemodynamic Values Following 120 Minutes of Treatment
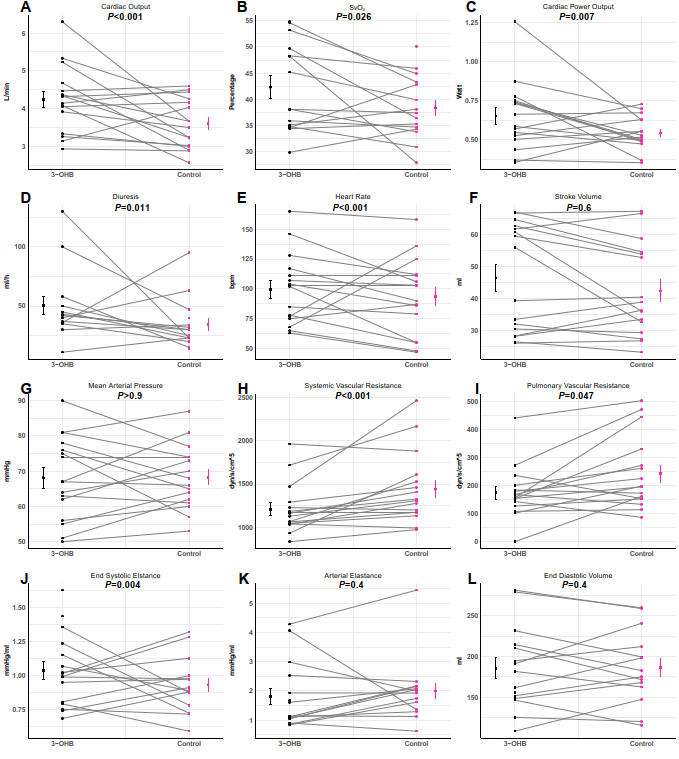


The mean of each hemodynamic value after 120 minutes of treatment with 3-OHB (black) and Control (pink) is indicated with a dot and error bars of ± SEM. Observations for each animals are represented with dots representing the value of the hemodynamic variable after 120 minutes of 3-OHB infusion (black dots) and Control infusion (pink dots). P-values indicate the statistical significance from the primary analysis.

**Supplemental Figure 3:** Response to Treatment Based on Intervention Sequence and Period

*
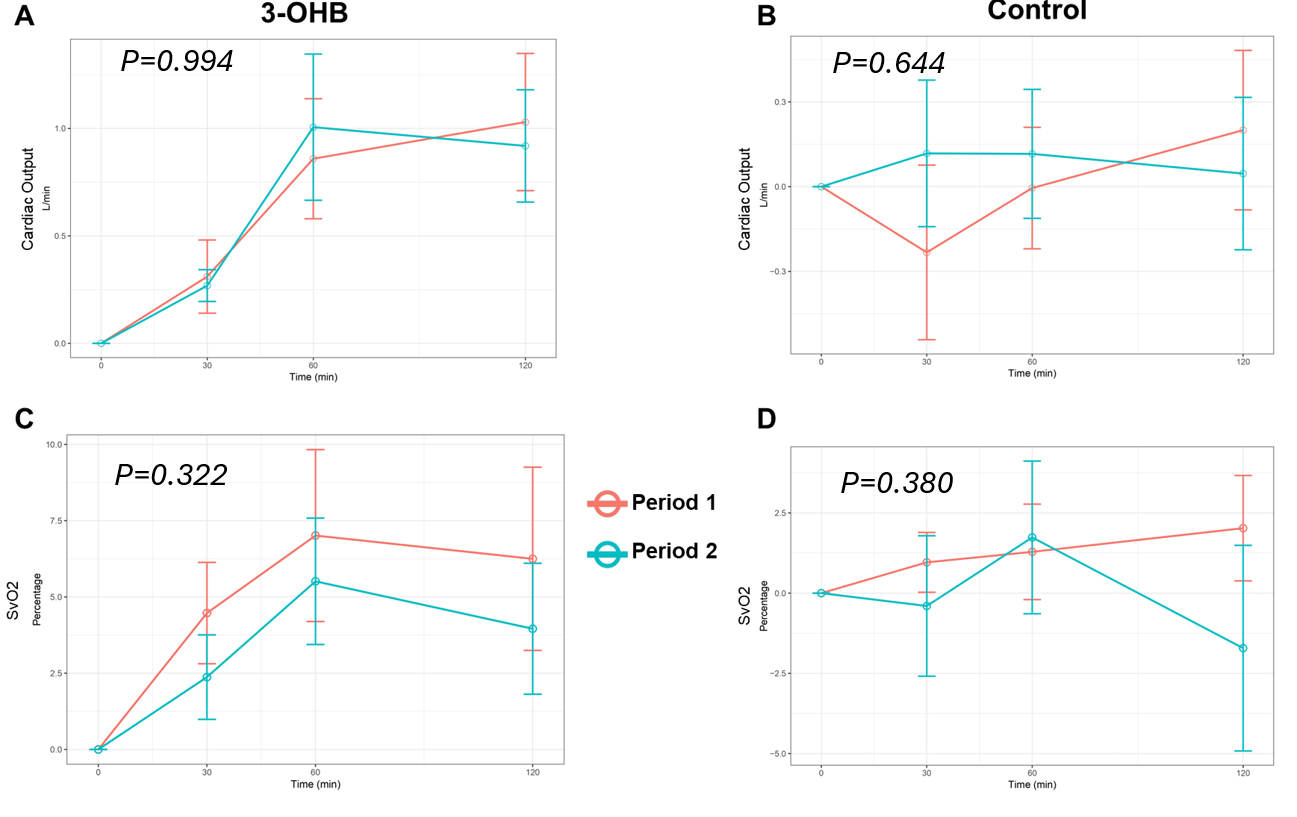
*

Top row: Change in cardiac output during treatment with 3-OHB (A) and control (B) for 120 minutes.

Bottom row: Change in mixed venous oxygen saturation (SvO2) during treatment with 3-OHB (C) and control (D).

The red lines represent the animals receiving the corresponding treatment (3-OHB or Con-trol) in the first infusion period. The blue lines represent the animals receiving the corre-sponding treatment in the second infusion period. The same number of animals were in each group (n=8).

Data are mean ± SEM.

**Supplemental Figure 4:** Changes in Mitochondrial Function During the Study Period


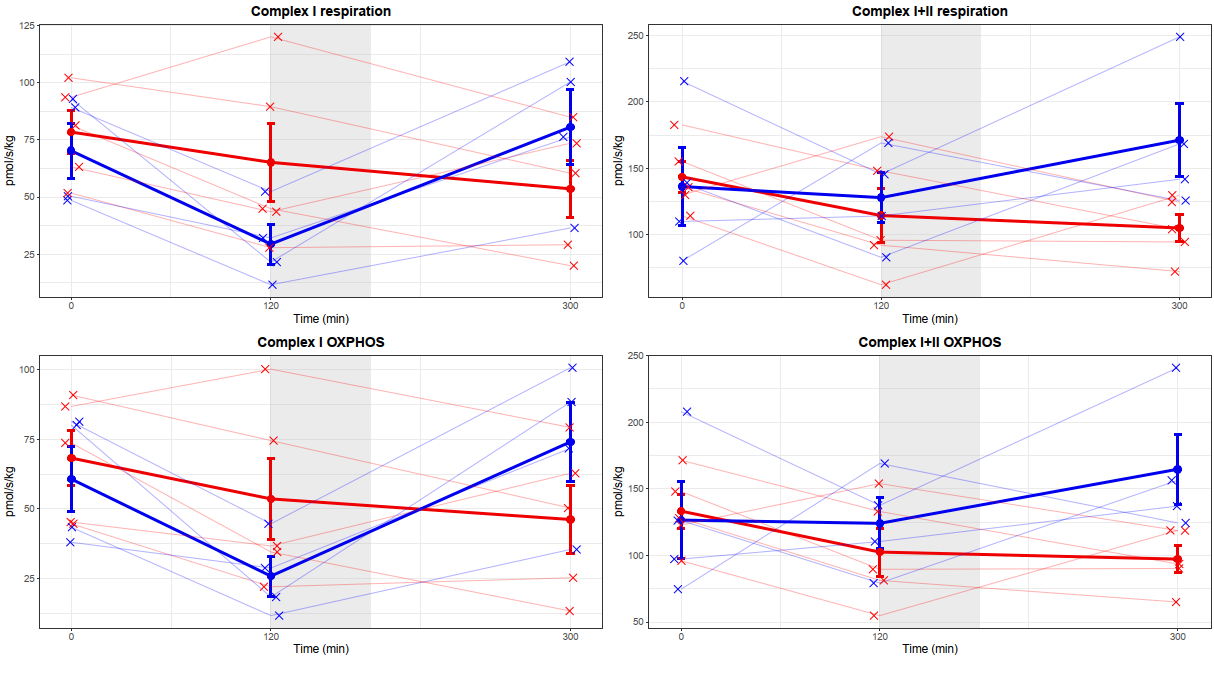


**3-OHB 🡪 Control Control 🡪 3-OHB**

Temporal evolution of mitochondrial function during infusion with 3-hydroxybutyrate (3-OHB) and the control infusion.

0 = shock state, 120 = end of first infusion, 180 = start of second infusion, 300 = end of second infusion.

Grey area marks the washout period where no intervention is given.

Red and blue crosses connected with lines represent each unique animal. The bold lines represent the mean ± SEM.

Biopsies were obtained in five animals randomized to 3-OHB 🡪 Control and in four ani-mals randomized to Control 🡪 3-OHB

**Supplemental Figure 5:** Key Temporal Evolution of Hemodynamics in Control Animals Underwent Endomyocardial Biopsy Sampling

Cardiac output, stroke volume, mean arterial blood pressure and cardiac power output in healthy state, right after onset of cardiogenic shock (CS) following embolization, after 60 minutes of no-touch (Shock) and for 180 minutes in animals receiving control infusion in the first intervention period undergoing myocardial biopsies(n=4). Endomyocardial biopsies were obtained at three timepoints (red vertical lines): right before start of the first infusion, right after the end of first infusion and right after the end of second infusion (not shown). The figure demonstrates that we observed no impact on cardiac function in from the biopsies before the second infusion period. Note that biopsies were not obtained at the start of the second infusion period. Black line is mean ± SEM. Each animal is displayed as pink lines.

**Supplemental Figure 6:** Time Course of hs-TnI release

**
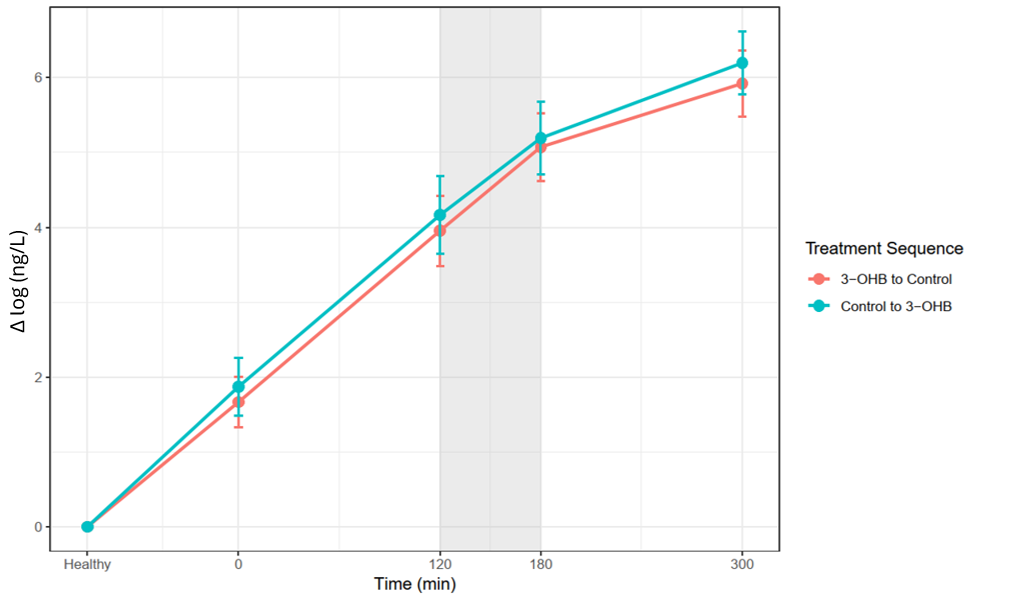
**

Temporal evolution of arterial plasma levels of high sensitivity troponin I (hs-TnI). Data are mean ± SEM. Grey area is the washout period, where no intervention was given.

**Supplemental Table 1:** Hemodynamic Changes Five Minutes after Embolization

| *N=16* | **Baseline** | **Onset of CS** | **Start of first infusion (Shock)** | **Start of second infusion** | |
| --- | --- | --- | --- | --- | --- |
| **Hemodynamic parameters** | | | | |  |
| CO, L/min | 4.5 ± 0.9 | 2.8 ± 0.5 | 3.1 ± 0.8 | 3.6 ± 0.7 | |
| SvO_2_, % | 55.0 ± 8.1 | 37.7 ± 5.8 | 37.1 ± 6.5 | 38.2 ± 6.6 | |
| MAP, mmHg | 84 ± 15 | 70 ± 11 | 68 ± 10 | 67 ± 11 | |
| CPO, W | 0.85 ± 0.26 | 0.43 ± 0.13 | 0.47 ± 0.18 | 0.55 ± 0.15 | |
| PaPi | 3.3 (2.3; 4.3) | 2.7 (2.3; 3.0) | 2.3 (1.5; 3.3) | 2.8 (1.9; 4.3) | |
| SV, mL | 76 ± 15 | 52 ± 13 | 48 ± 22 | 44 ± 18 | |
| HR, bpm | 63 (53; 68) | 51 (49; 58) | 68 (56; 74) | 85 (72; 111) | |
| mPAP, mmHg | 19 ± 5 | 20 ± 4 | 22 ± 5 | 23 ± 6 | |
| RAP, mmHg | 6 ± 3 | 7 ± 3 | 7 ± 3 | 6 ± 3 | |
| PAWP, mmHg | 8 ± 3 | 12 ± 3 | 14 ± 4 | 14 ± 5 | |
| SVR, dyn*cm^5^ | 1413 ± 291 | 1883 ± 508 | 1695 ± 495 | 1410 ± 440 | |
| PVR, dyn*cm^5^ | 204 ± 101 | 225 ± 146 | 210 ± 495 | 220 ± 93 | |
| P(v-a)CO_2_, kPa | 1.39 ± 0.32 | 1.68 ± 0.34 | 1.66 ± 0.36 | 1.62 ± 0.27 | |
| **Pressure-volume parameters** | | | | |  |
| LVESV, mL | 109 ± 32 | 131 ± 33 | 115 ± 33 | 125 ± 33 | |
| LVESP, mmHg | 99 ± 14 | 89 ± 21 | 84 ± 16 | 82 ± 13 | |
| LVEDV, mL | 190 ± 39 | 185 ± 36 | 172 ± 42 | 179 ± 46 | |
| LVEDP, mmHg | 15 ± 6 | 19 ± 6 | 20 ± 7 | 21 ± 9 | |
| LVEF, % | 50 (37; 64) | 33 (26; 43) | 36 (33; 43) | 39 (33; 47) | |
| Ees, mmHg/mL | 1.45 ± 0.56 | 0.97 ± 0.33 | 1.11 ± 0.31 | 1.01 ± 0.33 | |
| Ea, mmHg/mL | 1.66 ± 0.91 | 2.39 ± 1.40 | 1.92 ± 0.93 | 2.15 ± 1.60 | |
| Ea/Ees ratio | 1.42 ± 1.00 | 2.58 ± 1.45 | 1.89 ± 0.68 | 1.88 ± 1.02 | |
| Cardiac mechanical efficiency, % | 60 (50;70) | 42 (34; 49) | 44 (42; 55) | 49 (41; 55) | |

Data are expressed as mean ± SD or median (interquartile range). Hemodynamic parameters and pressure-volume parameters in healthy state, five minutes after onset of cardiogenic shock, at shock (start of the first infusion) and at the start of the second infusion. Below the absolute variable values the relative change compared with healthy baseline is stated.

CO=cardiac output, CPO=cardiac power output, PaPi=pulmonary artery pulsatile index, HR=heart rate, SV=stroke volume, SvO_2_=mixed venous saturation, P(v-a)CO_2_=veno-arterial carbon dioxide difference, MAP=mean arterial pressure, mPAP=mean pulmonary artery pressure, RAP=right atrium pressure, PAWP=pulmonary artery wedge pressure, SVR=systemic vascular resistance, PVR=pulmonary vascular resistance, Ea=arterial elastance, Ees=end- systolic elastance (the slope of the end-systolic pressure-volume relationship (ESPVR)), LVESV=end systolic volume, LVEDV=end diastolic volume, LVEF=left ventricular, ejection fraction.

**Supplemental Table 2:** Mitochondrial Function Following 120 Minutes of Treatment

|  | **Mean after 120 minutes of infusion** | |  |
| --- | --- | --- | --- |
|  | **3-OHB** | **Control** | ***P-*value** |
| Complex I respiration, pmol/s/kg | 76.3 (43.6 to 100.3) | 32.1 (21.5 to 60.4) | **0.008** |
| Complex I+II respiration, pmol/s/kg | 141.6 (95.5 to 168.3) | 113.8 (94.2 to 129.5) | 0.239 |
| Oxphos capacity complex I, pmol/s/kg | 71.7 (35.4 to 88.4) | 28.7 (18.3 to 50.3) | **0.005** |
| Oxphos capacity complex I+II, pmol/s/kg | 133.0 (89.6 to 153.9) | 110.4 (90.2 to 118.8) | 0.204 |

Mitochondrial function following 120 minutes of infusion with 3-hydroxybutyrate (3-OHB) or control. N=9. Data are expressed as median (interquartile range).

**Supplemental Table 3:** Comparison of Mitochondrial Function within Groups Depending on Treatment Period

|  | **3-OHB** | | | **Control** | | |
| --- | --- | --- | --- | --- | --- | --- |
|  | **End of period 1 (n=5)** | **End of period 2 (n=4)** | ***P-*value** | **End of period 1 (n=4)** | **End of period 2 (n=5)** | ***P-*value** |
| Complex I respiration, pmol/s/kg | 65.2±38.3 | 80.6±32.5 | 0.544 | 53.6±28.1 | 29.4±17.4 | 0.178 |
| Complex I+II respiration, pmol/s/kg | 114.1±45.5 | 171.2±55.0 | 0.132 | 127.7±37.6 | 104.7±23.4 | 0.297 |
| OXPHOS complex I, pmol/s/kg | 53.6±32.7 | 74.1±28.4 | 0.356 | 25.8±7.2 | 46.2±27.0 | 0.219 |
| OXPHOS complex I+II, pmol/s/kg | 102.5±40.2 | 164.6±52.4 | 0.083 | 124.1±38.3 | 97.3±22.4 | 0.228 |

Mitochondrial function following 120 minutes of infusion with 3-hydroxybutyrate (3-OHB) or Control.

Biopsies were performed in 5 animals receiving 3-OHB 🡪 Control and in 4 animals receiving Control 🡪 3-OHB.

Period 1 is the first 120 minutes before crossover.

Period 2 is the 120 minutes after crossover and one hour washout.

The *P*-value is derived from an unpaired t-test comparing the mitochondrial function within treatment groups (3-OHB or Control) depending on treatment periods.

**Supplemental Video 1:**

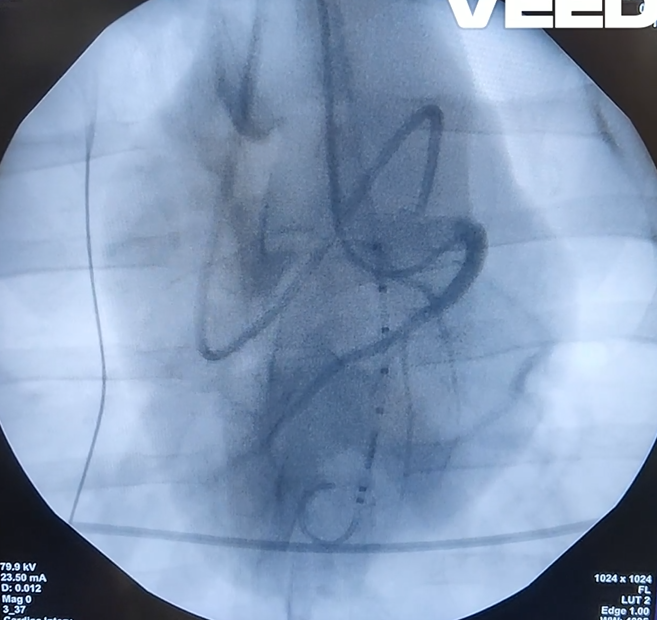


Video showing the catheterization of the cardiac chambers including the pulmonary artery catheter, left ventricle pressure-volume catheter and the left main coronary artery cathteter. The video shows one injection of microspheres using fluoroscopic verification.
